# Redefining an epitope of a malaria vaccine candidate, with antibodies against the N-terminal MSA-2 antigen of *Plasmodium* harboring non-natural peptide bonds

**DOI:** 10.1007/s00726-013-1541-x

**Published:** 2013-07-09

**Authors:** José Manuel Lozano, Yuly Andrea Guerrero, Martha Patricia Alba, Liliana Patricia Lesmes, José Oswaldo Escobar, Manuel Elkin Patarroyo

**Affiliations:** 1 Biocatalysis Department, Fundación Instituto de Inmunología de Colombia (FIDIC), Universidad del Rosario, Carrera 50 No. 26-20, 020304 Bogotá DC, Colombia; 2 Universidad Nacional de Colombia, Bogotá DC, Colombia

**Keywords:** Site-directed design, Peptide-bond isostere, Peptide mimetic, Antibody, Passive immunization, Malaria vaccine candidate

## Abstract

The aim of obtaining novel vaccine candidates against malaria and other transmissible diseases can be partly based on selecting non-polymorphic peptides from relevant antigens of pathogens, which have to be then precisely modified for inducing a protective immunity against the disease. Bearing in mind the high degree of the MSA-2^21–40^ peptide primary structure’s genetic conservation among malaria species, and its crucial role in the high RBC binding ability of *Plasmodium falciparum* (the main agent causing malaria), structurally defined probes based on non-natural peptide-bond isosteres were thus designed. Thus, two peptide mimetics were obtained (so-called reduced amide pseudopeptides), in which naturally made amide bonds of the ^30^FIN^32^-binding motif of MSA-2 were replaced with ψ–[CH_2_–NH] methylene amide isostere bonds, one between the F–I and the second between I–N amino acid pairs, respectively, coded as ψ-128 ψ-130. These peptide mimetics were used to produce poly- and monoclonal antibodies in *Aotus* monkeys and BALB/c mice. Parent reactive mice-derived IgM isotype cell clones were induced to Ig isotype switching to IgG sub-classes by controlled in vitro immunization experiments. These mature isotype immunoglobulins revealed a novel epitope in the MSA-2^25–32^ antigen and two polypeptides of rodent malaria species. Also, these antibodies’ functional activity against malaria was tested by in vitro assays, demonstrating high efficacy in controlling infection and evidencing neutralizing capacity for the rodent in vivo malaria infection. The neutralizing effect of antibodies induced by site-directed designed peptide mimetics on *Plasmodium*’s biological development make these pseudopeptides a valuable tool for future development of immunoprophylactic strategies for controlling malarial infection.

## Introduction

Malaria, one of the most important public health problems worldwide, is a lethal infectious disease that is currently responsible for about 300–500 million clinical cases and more than 2 or 3 million deaths per year, mainly among children in developing countries. Malaria is caused by the *Plasmodium* protozoan which is transmitted to vertebrates by the bite of a female *Anopheles* mosquito. The parasite’s asexual blood forms (merozoites and schizonts) are the life-cycle stages which are responsible for *Plasmodium* infection morbidity and mortality in the vertebrate host (Phillips 2001; World Malaria Report 2010 2011; Singh et al. 2004; Tuteja 2007; Hay et al. 2004). Most deaths caused by malaria are due to *Plasmodium falciparum*, the disease’s most aggressive causative agent (World Malaria Report 2010 2011; Singh et al. 2004; Tuteja 2007; Hay et al. 2004; Parham and Michael 2010; Paaijmans et al. 2010; D’alessandro et al. 1995).

Developing a totally effective antimalarial vaccine has thus become a major challenge for biomedical research to control this deadly disease. Current vaccine development has focused on multi-component, multi-stage, subunit-based synthetic vaccines because obtaining an appropriate anti-malarial immune response depends on ensuring that several peptides can work together to attack different parasite targets, in combination with improved delivery systems, as the parasite cannot be regarded as a passive target but one which dynamically adapts to any attack made by the immune system while it is invading and living within the host. On the other hand, growing parasite resistance to chloroquine and other antibiotics used for its control is becoming a non-manageable worldwide problem. Due to all the above, finding new strategies for controlling malaria has become an urgent need from both immunoprophylactic and immunotherapeutic perspectives for preventing the action of this deadly parasite.

A number of parasite molecules have been tested as possible vaccine candidates; among them, the merozoite surface antigen-2 (MSA-2 or MSP-2) is an attractive target for developing new molecular tools against malaria. This is one of the most abundant and highly polymorphic non-structured antigens being synthesized in both trophozoite and schizont blood stages of *Plasmodium* as a precursor of 274 amino acids with an estimated relative molecular weight of 28.4 kDa. This antigen is anchored to the parasite surface membrane through a tail of glycosylphosphatidylinositol (GPI) (Eisen et al. 1998). According to different reports, this surface antigen has been characterized as having a relative molecular mass ranging from 30 to 45 kDa (Adda et al. 2009; Smythe et al. 1990) and is constituted by two genetically conserved regions, one located at the C-terminus and the other at the N-terminus. It also contains a polymorphic region and two semi-conserved regions located at the center of the antigen’s primary structure, allowing two different allelic families; thus, the MSA-2 exact molecular mass still remains a controversial matter. Given the relevance of *Plasmodium* survival based on this antigen and bearing in mind that people naturally exposed to malaria produce high levels of antibodies against the N-terminus portion of MSA-2 and such humoral response has been associated with protection against this lethal disease, we propose a site-directed non-naturally modified N-terminus peptide sequence of MSA-2 as an important target for functional antibody induction.

With the aim of analyzing the functional role of the MSA-2^27–34^ epitope (^27^SNTFINNA^34^) reported by Jones et al. (1996), which was also identified by Ocampo et al. (2000) on a high-activity binding peptide (HABP) coded as 4044(^21^KNESKYSNTFINNAYNMSIR^40^) that binds to red blood cells (RBCs) in a highly specific fashion, a bioinformatics analysis was performed using both the entire MSA-2 primary sequence and the one of peptide 4044 located at the MSA-2 N-terminus portion (MSA-2^21–40^). On the other hand, a reading register for binding an N-terminus MSA-2^21–40^ (peptide 4044) to pockets 1–9 of the HLA-DRβ1 molecule was determined between residues F^30^ and S^38^ according to data reported by Patarroyo et al. (2011). The presence of this highly conserved fragment among different *Plasmodium* species has led to developing pseudopeptide solid-phase synthesis. As denoted by the underlined residues (shown above), 4044 has three RBC-binding motifs. The ^30^FIN^32^-binding motif located at the central portion of the molecule was chosen as the basis for our experimental design.

Based on the information described above, a decision was made to maintain the 4044 primary structure intact, but modify specific target peptide bonds in the ^30^FIN^32^ or ^30^Phe-Ile-Asn^32^ peptide sequence to produce two reduced amide pseudopeptide analogs, each harboring only one substituted peptide bond. Both peptide mimetics were thus obtained as monomer and polymer forms, in agreement with a peptide-bond modification strategy, based on a site-directed design. The so-obtained new molecules, derived from parent peptide 4044, were coded as ψ-128 (^31^Ile-Asn^32^) and ψ-130 (^30^Phe-Ile^31^), with their polymer forms being coded as ψ-129 and ψ-131, respectively. The present work was thus aimed at presenting evidence on the impact of introducing a modified peptide bond and its implications in the molecule’s secondary structure, immunogenic capacity and ability to induce functional antibodies able to neutralize malarial infection. These isotype-defined antibodies’ immunotherapeutic in vitro and in vivo potential was assessed in the BALB/c mouse animal model by passively transferring specific immunoglobulins to mice challenged with lethal doses of both *Plasmodium berghei* and *Plasmodium yoelii* strains in parallel assays. As demonstrated here, peptide-mimetic-induced immunoglobulins were able to neutralize malarial infection and so constitute a novel immunotherapeutic tool to be further considered in more complex animal models and may also be potentially useful for controlling human malaria.

However, designing both 4044-derived peptide mimetics represented a chemical challenge because, as recently reported by our group, determining pseudopeptide sequences containing isostere peptide bonds, either on asparagine (Asn) or glutamine (Gln) residues, can make synthesizing difficult since such precursor amino acid aldehydes are obtained in lower than 0.5 % yields. We have thus recently proposed a novel strategy for obtaining both Asn and Gln aldehyde synthons based on a controlled side-chain protection approach, as well as a suitable solvent partition procedure affording yields higher than 80 % (Carreño et al. 2011). The analog coded ψ-128, harboring a reduced amide bond located just between the Ile-Asn amino acid pair, represented an experimental challenge in the present work. Remarkably, by using the above-mentioned strategy both designed ψ-128 (^31^Ile-Asn^32^) and ψ-130 (^30^Phe-Ile^31^) peptide mimetics were successfully obtained in normal yields, as discussed below.

Monoclonal antibodies raised against both 4044 pseudopeptides reveal a novel epitope within the MSA-2^21–40^ primary structure; this is constituted by the sequence ^25^KYSNTFIN^32^ at the N-terminus of MSA-2, which overlaps portions of the former epitope reported by Jones and the high-activity binding peptide HABP coded 4044, reported by Ocampo (Ocampo et al. 2000). Remarkably, these antibodies induced by well-defined molecular structural probes cross-react with polypeptides, showing two strong bands at relative molecular weights ranging between 50.11 and 63.09 kDa in the cytosol of *P. berghei* ANKA and also two strong bands in a molecular weight range from 44.46 to 50.11 kDa in the cytosolic extract of *P. yoelii* 17XL. As demonstrated, when passively transferred, these monoclonal antibodies controlled experimental malaria in a rodent animal model. Thus, these novel kinds of molecular tools constituted by site-directed designed pseudopeptides and their induced antibodies possess a high potential for future application in controlling human malaria.

## Materials and methods

### Experimental design for obtaining site-directed peptide bond modifications on parent peptide 4044

Introducing peptide bond surrogates (denoted ψ[…], according to Spatola’s nomenclature for a class of peptide mimetics called pseudopeptides (Spatola 1983), has been long recognized as a powerful strategy for enhancing peptides’ biological lifetime, improving biological activity or selectivity, designing enzyme inhibitors or inducing particular conformational features in potential vaccine candidates. Solid-phase synthesis for reduced amide pseudopeptides has become an easy procedure for obtaining novel structure-defined molecules.

The experimental design was based on previously reported binding motifs present in parent peptide 4044 amino acid sequence (^21^KNESKYSNTFINNAYNMSIR^40^), derived from the MSA-2 antigen, which has been previously described by Ocampo et al. (2000). As denoted by the underlined residues, this sequence has three high RBC-binding motifs, two of them being located toward the N-terminus and the other at the center of the sequence. The latter was selected for the molecular design of two site-directed peptide bond modifications, due to the potential role as B-epitopes by inducing a humoral immune response by specific antibody stimulation. As mentioned above, previous work by Jones has reported the ^27^SNTFINNA^34^ amino acid sequence, located at the MSA-2 surface antigen N-terminus, as a highly immunogenic epitope (Jones et al. 1996). For all the reasons described above, the ^30^FIN^32^ amino acid sequence was taken as the target for our molecular peptide-mimetic design for modifying peptide bonds between the ^30^Phe-Ile^31^ and ^31^Ile-Asn^32^ amino acid pairs to afford the reduced amide pseudopeptide coded as ψ-128 and its ψ-129 polymer form, as well as the ψ-130 analog and its ψ-131 polymer form, respectively as described below.

### Bioinformatic analysis

Amino acid sequences from *Plasmodium* MSA-2 superfamily were used as input for the bioinformatic analysis. All sequences were obtained from the *Plasmodium* Genomics Resource database *PlasmoDB* (http://plasmodb.org/plasmo/); the MSA-2 *P. falciparum* 3D7 amino acid sequence had the gene bank PFB0300c accession number. Subsequently, a protein *blast* analysis for a multiple sequence alignment was carried out (http://blast.ncbi.nlm.nih.gov/Blast.cgi) against the rodent malaria species *Plasmodium berghei* and *Plasmodium yoelii* genome-reported proteins. Thus, 11 hypothetical *P. yoelii* proteins matched the MSA-2 sequence, while 5 hypothetical proteins were found on *P. berghei*. On the other hand, the MSA-2^21–40^ amino acid sequence was also used for *blast* analysis as above to assess the HABP-specific primary structure. As a result, the entire or partial amino acid sequence of the MSA-2^21−40^ (peptide 4044) was found in 56 hypothetical, putative and expressed proteins of *P. yoelii* and 44 polypeptides of *P. berghei*, respectively.

### Solid-phase synthesis and characterization of ψ–[CH_2_–NH] reduced amide peptide mimetics

Reduced amide pseudopeptide synthesis involves both liquid and solid phase steps. The aldehyde form for the carboxylic acid function is obtained during the first step; after this,* t*-Boc–*N*-protected amino acid carboxamide is transformed into a formyl function, carried out for the amino acid located at the *N*-site of the bond needed to be modified. The second step consists of condensation of the above *t*-Boc–amino acid aldehyde to the unprotected amino acid of the growing peptide chain, located at the *C*-site of the bond to be modified. This procedure is carried out using the strategy originally reported by Fherentz and Castro (1983).

Briefly, the standard procedure for reducing the carbonyl function of an amino acid carboxamide intermediate derivative of a *t*-Boc-protected amino acid involves a strong reduction of this carboxamide with LiAlH_4_ in dry ether. This procedure affords global yields of about 80 % for most amino acids. In spite of being a chemically clean procedure producing few non-desirable subproducts, physicochemical characterization of the obtained product is recommended, as well as that for its starting amino acid precursor and its corresponding carboxamide derivative by using FT-IR, thin-layer chromatography (TLC) and unidimensional ^1^H-NMR.

*t*-Boc–Phe and *t*-Boc–Ile aldehyde forms were condensed to their corresponding growing peptide resin and a second mild reduction step with NaCNBH_3_ was carried out for each adduct to simultaneously obtain all target molecules, coded ψ-128 (F–ψ–[CH_2_NH]–I), ψ-129 (F–ψ–[CH_2_NH]–I, polymer form), ψ-130 (I–ψ–[CH_2_NH]–N) and ψ-131 (I–ψ–[CH_2_NH]–N, polymer form).

All molecules were manually synthesized by *tert*-butyloxycarbonyl (*t*-Boc)-based solid-phase peptide synthesis (SPPS), following a protocol first reported by Merrifield (1963) and later modified for multiple peptide synthesis (Houghten 1985). Each amino acid residue was placed on the pseudopeptide backbone, as described elsewhere (Cushman et al. 1991; Sasaky et al. 1987). The ψ–[CH_2_NH] surrogate was introduced by deprotected *N* ^α^-amino group resin-bound reductive alkylation with the *t*-Boc-protected amino acid aldehyde (0.576 mmol) in DMF containing 0.5 % acetic acid (HOAc), followed by portion-wise addition of NaBH_3_CN (0.670 mmol) for 40–60 min. Total coupling was checked by the ninhydrin test and the coupling reaction was repeated when necessary. Coupling was allowed to proceed for 5 h with constant shaking, followed by several washes with *N,N*′-dimethylformamide (DMF), isopropanol and dichloromethane. Standard solid-phase peptide synthesis was carried out to introduce the remaining *t*-Boc amino acids to the last N-terminal residue. Protected pseudopeptide-resin batches were treated with trifluoroacetic acid (TFA) and cleaved from the resin by treatment with low concentrations of anhydrous hydrogen fluoride (HF), containing 10 % anisole at 0 °C for 60 min. After HF evaporation in an N_2_ stream, each pseudopeptide-resin product was washed with cold diethyl ether, then extracted with 5 % HOAc and finally lyophilized. The raw products obtained for each ψ–[CH_2_NH] surrogate were further analyzed by analytical RP-HPLC, purified by preparative RP-HPLC and identified by MALDI-TOF mass spectrometry.

Polymer forms for both reduced amide pseudopeptides (coded ψ-129 and ψ-131) had identical amino acid sequences regarding their corresponding monomers ψ-128 and ψ-130, but were different regarding their N- and C-terminals, the polymer forms containing a Cys-Gly pair of residues at the N-end and a Gly-Cys pair of residues at the C-terminus. After being cleaved from the resin, these polymer forms were dissolved in isotonic saline solution at 4 mg/mL concentration and bubbled in an N_2_ stream at room temperature, pH 7.4, until no thiol free groups were detected. This procedure was carried out using the Elman reaction, proving that all sulfhydryl groups were involved in the intermolecular disulfide bridges. Table 1 shows the main physical and chemical characteristics for all molecules.

**Table 1:** Physical and chemical characteristics of the peptide MSA-2^21−40^ and its pseudopeptide analogs

Code	Amino acid sequence	Theoretical MW (Da)	Experimental *m*/*z* [M + H^+^]
4044	KNESKYSNTFINNAYNMSIR	2,388.01	2,386.05
4306	CGKNESKYSNTFINNAYNMSIRGC	2,715.50	2,713.13
ψ-128	KNESKYSNTF–ψ[CH_2_–NH]–INNAYNMSIR	2,379.14	2,380.03
ψ-129	CGKNESKYSNTF–ψ[CH_2_–NH]–INNAYNMSIRGC	2,706.64	2,710.31
ψ-130	KNESKYSNTFI–ψ[CH_2_–NH]–NNAYNMSIR	2,379.14	2,380.09
ψ-131	CGKNESKYSNTFI–ψ[CH_2_–NH]–NNAYNMSIRGC	2,706.64	2,704.26

Peptide mimetics were obtained in average yields of about 70 % and their guaranteed purity was higher than 99 %, as judged by RP-HPLC. Their identity was assessed by MALDI-TOF mass spectroscopy. Monomer forms for each target molecule were also characterized by uni- and bi-dimensional ^1^H-NMR techniques, as will be discussed below.

### Thin-layer chromatography (TLC)

Thin-layer chromatography was used to assess* t*-Boc-amino acid aldehyde formation on silica gel pre-coated aluminum plates (F_254_ silica gel 60, Merck, Darmstadt, Germany), using some of the following solvent systems: EtAcO-*n*-hexane (2:1), EtAcO-*n*-hexane (1:1) or a solvent system based on CHCl_3_–MeOH (2:1), as suitable. Amino acid derivatives were analyzed by fluorescence at short UV wavelength (254 nm), or ninhydrin by a follow-up reaction.

### Fourier transformed-infrared spectroscopy (FT-IR)

Infrared spectra for all *N*-α-*t*-boc–Asn and *N*-α-*t*-boc–Gln side-chain protected amino acids and their derivatives were recorded on a Jasco FT/IR-460 plus spectrophotometer (Tokyo, Japan). Samples were analyzed on a PIKE MIRacle AG single reflection horizontal ATR cell (Pike Technologies, Madison, WI, USA) by dispersing either a lyophilized compound or its suspension, subjected to 21 psi (pounds/square inch) pressure. Spectra were recorded after 64 scans at 4 cm^−1^ resolution.

### Mass spectrometry analysis by MALDI-TOF

Mass spectra were recorded on a Bruker Protein TOF mass spectrometer with reflectron mode (Billerica, MA, USA). MALDI (matrix-assisted laser desorption ionization) experiments were performed using the TOF (time of flight) technique. This instrument uses an N_2_ laser, radiating at 337-nm wavelength with 3-ns pulses. The acceleration voltage was +17.5 kV and reflectron voltage was +20 kV. All spectra were obtained by a respective series of ten laser pulses to ensure comparable conditions. Laser power was as minimal as possible for each measurement. The matrix used in this work was α-cyano-4-hydroxycinnamic acid (CCA) (Sigma Chemical Co., Saint Louis, MO). The CCA matrix was prepared as a saturated solution in 1 mL TA (40 % acetonitrile in 0.1 % trifluoroacetic acid). Samples were dissolved in TA to give a 100 pmol/μL concentration. Samples were prepared for MALDI-TOF analysis by diluting the sample solution in the matrix-saturated solution to reach a 10 pmol/μL concentration. Then, 0.5 μL aliquots of sample-matrix mixture was poured onto the target plate, air-dried and analyzed.

### Circular dichroism (CD) experiments

Circular dichroism (CD) assays were carried out at room temperature on nitrogen-flushed cells using a Jasco J-810 spectropolarimeter (Madrid, Spain). Spectra were recorded within a 190–260-nm wavelength interval, using a 1-mm path-length rectangular quartz cell. Each spectrum was obtained from averaging three scans taken at a 20 nm/min scan rate with 1-nm spectral bandwidth corrected for baseline deviation using Jasco software. The CD profile for each molecule was obtained by dissolving lyophilized purified peptides in water or 30 % aqueous 2,2,2-trifluoroethanol (TFE), 0.5 mL final volume. A typical 0.2 mM peptide/pseudopeptide concentration in TFE–water mixture was stabilized but did not induce secondary structure in peptides, as described elsewhere (Lozano et al. 2007). The results were expressed as mean residue ellipticity (θ), the units being deg × cm^2^ × dmol^−1^ according to the *(θ)* = *θ*_*λ*_ */(100lcn)* function, where *θ*_*λ*_ is measured ellipticity, *l* the optical path length, *c* peptide/pseudopeptide concentration and *n* the number of amino acid residues in the sequence of interest.

### Secondary structure analysis by proton nuclear magnetic resonance (^1^H-NMR)

For NMR measurements, 10 mg of each lyophilized peptide or pseudopeptide analog was dissolved in a H_2_O/D_2_O mixture (9:1 ratio), as well as in an aqueous 30 % TFE solution (99.94 % D 2,2,2-trifluoroethanol-d3, Cambridge Isotope Laboratories, Andover, MA, USA) until reaching a final 0.5 mL volume. Proton nuclear magnetic resonance (^1^H-NMR) spectra were recorded on a Brucker DRX 600 MHz spectrophotometer, provided with a temperature control unit. Spectra were recorded between 280 and 310 K using 4.5–5.0 pH range and referenced to water’s internal signal at 4.75 ppm. Routine COSYGSmtprtp (Avance version, Phase Sensitive) was performed to assign all spin systems, using gradient pulses for detection with multiple-quantum filter, according to gradient-radio experiments. TOCSY mievgstp 19 (Avance version, Homonuclear Hartman-Hahn Transfer), using mLEV 17 sequence mixing and 80–100 ms mixing-time, was carried out to corroborate NH and C^α^H side-chain connectivities for each spin system. Experiments (Advance version, 2D-Homonuclear Correlation via Dipolar Coupling Phase Sensitive using TPPI) were carried out for 150, 200–300, 400 and 500-ms mixing times to assign all sequential neighbors for the NOESY gstpi9-peptide chain. The 2D data were processed on a Silicon Graphics-Brucker Indy computer provided with XWINNMR 1.3 software (Bruker, Darmstadt, Germany).

Temperature coefficients were determined from TOCSY spectra using a 285–315 K temperature range. A slope was deduced from a linear relationship established for chemical shift *cf* temperature pattern for each hydrogen from the amide groups (−ΔδHN/ΔT, ppm/K). ^3^J_HNHα_ coupling constants were measured from the separation of multiplets in cross-peaks from unidimensional DFQ-COSY experiments.

### Structural calculation

The structural calculation was performed by using the software provided by Accelrys in Silicon graphics workstations. NOE peaks, selected from 400 ms NOESY data sets, were integrated and converted into distance restraints. These restraints were grouped as strong, medium and weak corresponding to 1.8–2.5 Å, 2.5–3.5 Å and 3.5–5.0 Å distance restraints, respectively. Distance Geometry (DGII) software was used for producing 50 starting structures for each analyzed molecule, in this case peptide 4044 and its peptide mimetics ψ-128 and ψ-130. These resulting 50 conformers were molecularly refined using a strategy known as restricted molecular dynamic (rMD) as previously described (Havel et al. 1985).

### Producing poly- and monoclonal antibodies for ψ-128 (ψ-129) and ψ-130 (ψ-131) peptide mimetics and immunochemical tests

Polyclonal antibodies induced by 4044-reduced amide pseudopeptides were produced in *Aotus* monkeys after being administered in three groups of eight animals each with polymer forms of the native 4044 peptide and its reduced pseudopeptide analogs in a three-dose scheme, by formulating 250 μg of each immunogen dissolved in isotonic saline solution and emulsified with Freund’s Complete Adjuvant for the first administration and Freund’s Incomplete Adjuvant for the two boosts. Samples of blood from treated animals were obtained after the second and third administrations and sera samples were kept frozen until use for immunochemical experiments. Animals were maintained according to international and Colombian regulations for animal welfare in the FIDIC animal colony station located in the city of Leticia, Amazonas.

In parallel experiments, spleen cells from BALB/c mice, previously immunized with pseudopeptide polymers ψ-129 and ψ-131, were fused to murine X63Ag8 myeloma cells using polyethylene glycol 3,000–3,700 (Sigma Chemical Co., St. Louis, MO, USA), according to previously described methodology, to obtain reactive hybridomas, as originally proposed by (Kohler and Milstein 1976; Lozano et al. 2007). These hybridomas were cloned twice by limiting dilution in flat-bottomed 96-well plates; supernatants were assessed for specific antibody reactivity by ELISA and Western blot. Cell clones having specific reactivity were amplified to higher cell culture volume.

### ELISA

Enzyme-linked immunosorbent assays (ELISA) were carried out for detecting antibody presence and measuring Ig isotypes pools after being purified. Briefly, each ELISA microplate well (Nunc, Inter, Med, Denmark) was coated with 100 μL of 0.005 μg/μL pseudopeptide as well as with *P. falciparum* lysate dissolved in bicarbonate buffer, pH 9.63. 100 μL samples of pure Ig pools (diluted 1:100 at an average concentration of 0.4 μg/mL) were tested in duplicate after blocking wells with 3 % skimmed milk in 0.05 % PBS-Tween-20. Specific anti-tested compound activity was detected using a 1/1,000 goat anti-rabbit IgG (H + L) peroxidase conjugate (Vector Laboratories, Inc. Burlingame, CA, USA). A tetramethylbenzidine (TMB) developing solution (KLP, Gaithersburg, MD, USA) was used as the substrate. The reaction was stopped by adding 50 μL of 1 M H_3_PO_4_ per well. Results were expressed as OD values, obtained at 450 nm.

### Immunoblotting

The *P. falciparum, P. berghei and P. yoelii*-schizont lysates were dissolved in Laemmli’s buffer, using β-mercaptoethanol as reducing agent and resolved either on fixed 10 % or on 7.5–15 % gradient gels by SDS-polyacrylamide gel electrophoresis (Green 1978). The resolved proteins were electro-transferred to a nitrocellulose membrane (Smythe et al. 1988). The antibody Ig pools or monoclonal antibodies were used as primary antibodies and detected using goat anti-mouse IgG (H + L) alkaline phosphatase conjugate (Vector Laboratories, Burlingame, CA, USA). A BCIP/NBT solution (Promega, Madison, WI, USA) was used as reaction substrate.

### In vitro IgM switching to IgG isotype

A thymocyte-conditioned medium was prepared according to a previously reported strategy (Pardue et al. 1983) to stimulate lymphocyte and cytokine proliferation in the presence of either ψ-128 or ψ-130 antigen, respectively. BALB/c mice thymuses were surgically removed from 2- to 4-week-old animals; the thymus cells were then cultured for 48 h at 37.8 °C in 5 % CO_2_ in RPMI 1640 medium, containing 10 % FBS. The thymocyte-conditioned medium (TCM) was then harvested by centrifuging (1,500 rpm for 5 min to pellet the thymus cells) and the supernatant (TCM) was stored at −20 °C until use. Cell clones were originally coded as 1A8A8, induced by the ψ-130 (^30^F–I^31^), and the 1H10E5G6 clone was induced by ψ-128 (^31^I–N^32^) antibodies, secreted by both cell clones strongly cross-reacting with both native and denatured MSA-2 antigen from the human malaria *P. falciparum* FCB2 strain. Cells were dispersed at 100,000 cells per well in flat-bottomed 96-well plates. Cells were then pulsed seven times with 200 ng of either ψ-130 or ψ-128, previously diluted in RPMI 15 % FBS and 50 % TCM, at intervals of 8–15 days per pulse. Cell supernatants were periodically harvested to determine their antibody isotype by using a Monoclonal Antibody Isotyping Kit (HRP/ABTS) from Thermo Scientific (Rockford, IL, USA). ψ-128 (ψ-129)-induced clones were coded 6 and 7 and those induced by ψ-130(ψ-131) were coded G, M, O and Q.

### In vivo production of ψ-129 and ψ-131 peptidomimetic-induced monoclonal antibodies

Macrophages were stimulated in the peritoneal cavity of BALB/c mice by ip administration of 0.5 mL pristane (Sigma Chemical Co., Saint Louis, MO, USA) for scaling up the production of each monoclonal antibody. Eight days after each animal had received the ip injection, 1 × 10^6^ cells of cell clone were induced by both ψ-129 and ψ-131, suspended in a 0.5 mL RPMI-1640 base. Ten days after cell inoculation, the ascitic fluid so produced was collected by an abdominal puncture made with a sterile needle; this was then stored at −20 °C until use. When a sufficient amount of each fluid had been collected, the complement was deactivated for 30 min at 56 °C and then centrifuged at 800 rpm for 5 min at 4 °C; the lipid material was removed and subsequently immunoglobulin purified (Lozano et al. 2007).

### Immunoglobulin isolation and purification

Antibodies specific for each pseudopeptide were then selectively precipitated by saturation with 80 % ammonium sulfate to obtain anti-pseudopeptide specific antibodies from ascitic fluids and clone cell culture supernatants. After spinning, supernatants were carefully removed and precipitated antibodies were reconstituted using 10 mM Tris–HCl pH 8.5 and dialyzed against the same buffer on a Spectra-pore dialysis membrane (Houston, Texas, USA) at 4 °C.

Weak anionic exchange chromatography (AEC) was performed with the antibody-enriched dialyzed precipitates obtained from reactive rabbit sera using DEAE-Sephadex A-25 resin (Pharmacia, Uppsala, Sweden) previously equilibrated with 10 mM Tris–HCl buffer, pH 8.5. Antibodies were left to bind at 4 °C overnight with constant shaking. After the column had been appropriately packed, Igs were eluted from the column by progressively increasing the buffer’s ionic strength from 50, 100 to 500 mM NaCl. A total of four column volumes (250 mL) were added per step, collecting 10 mL fractions at a constant 0.5 mL/min flow rate. The resin was then re-equilibrated with 10 mM Tris–HCl buffer, pH 8.5. All elution fractions were characterized by direct Dot blot for Ig detection and positive fractions were submitted to indirect Dot blot against a *P. falciparum* lysate, as well as against each 4044 pseudopeptide. Reactive elution fractions from direct and indirect Dot blots were pooled, taking into account the ionic strength order in which they had been eluted. Pooled fractions were subsequently dialyzed against PBS at 4 °C. Each Ig pooled fraction’s purity was assessed by sodium dodecyl sulfate polyacrylamide gel electrophoresis (SDS-PAGE). Further characterization was performed to define Ig reactivity against a *P. falciparum* lysate by Western blot.

### Ig protein determination

Each Ig pool’s protein concentration was determined by using a micro BCA protein assay (Pierce, Rockford, Illinois, USA). Pure pooled Ig samples from the original stocks were diluted 1/10 to 1/100 times in isotonic saline solution and 200 μL of the reagent A (bicinchoninic acid) and B (Cu^2+^) mixture was then added, according to the manufacturer’s recommendations. Samples were incubated in duplicate at 37 °C for 30 min before optical density was measured (OD) at 570 nm. Each sample protein concentration was determined by comparing its absorbance against a calibration curve, obtained from 0 to 8 μg samples from a 2 mg/mL BSA standard diluted in PBS. The color complex was stabilized by adding 50 μL of 1 N NaOH.

### *P. falciparum* recombinant MSP-2 expression and purification

Portions of the human PfMSP-2 *P. falciparum* 3D7 protein coding gene (Pf3D7_02) was cloned in the pQE-30 expression vector, which adds a 6-histidine tail to its N-terminal region. The recombinant plasmid was transfected in E. coli RG and the strain was cultivated in Terrific Broth medium (TB) (12 g/L tryptone, 24 g/L yeast extract, 4 mL/L glycerol, 2.31 g/L KH_2_PO_4_ and12.54 g/L K_2_HPO_4_), supplemented with ampicillin (0.1 mg/L) and chloramphenicol (0.034 mg/L) for 12 h at 37 °C with constant shaking. 950 mL TB medium was then inoculated with continuous shaking at 37 8 °C until reaching 0.6–0.8 OD at 600 nm. Recombinant protein expression was induced by adding IPTG (isopropyl-β-d-thiogalactopyranoside) at 1 mM final concentration and incubating for 5 h at 37 °C. After centrifuging at 10,000 rpm for 30 min, the pellet was treated with denaturing agents (6 M urea, 10 mM Tris–HCl, 100 mM NaH_2_PO_4_ and 15 mM imidazole) and lysozyme (1 mg/mL). The sample was sonicated and centrifuged at 12,000 rpm for 35 min at 48 °C for collecting the supernatant and subsequently submitting it to SDS-PAGE and Western blotting to ascertain recombinant protein expression.

### In vitro malarial invasion inhibition by pseudopeptide-induced antibodies

Antibodies were tested for their ability to inhibit *P. falciparum* (FCB-2 strain) invasion of human RBCs in in vitro assays. Ring-stage-iRBCs (>5 % parasitemia) were synchronized using the sorbitol technique (Lambros and Vanderberg 1979) and incubated in complete RPMI 1640 media supplemented with 25 mM HEPES buffer, 1 mg/mL hypoxanthine, 40 μg/mL gentamicin, 5 U/mL penicillin, 2 g/L glucose, 5 % NaHCO_3_ and 10 % 0 + plasma. When parasites had reached the schizont stage, 96-well plates containing three antibody dilutions (1:2, 1:4 and 1:8) were seeded with cultured iRBCs and additional non-iRBCs for completing a final 100 μL volume per well at 1.5 % hematocrit and 0.3 % parasitemia. All antibodies were assessed in triplicate, being incubated for 18 h at 37 °C in 5 % O_2_, 5 % CO_2_, and 90 % N_2_. Cells were harvested after centrifugation and 50 μL supernatant was removed and replaced by 100 μL, 15 μg/mL hydroethidine. The pellet was resuspended in 300 μL PBS following further incubation at 37 °C for 30 min and washed with PBS, then poured into polystyrene tubes and quantified for parasitemia by flow cytometry using a FCAScan equipment (Becton–Dickinson, San José, CA, USA). The sequence of events was recorded and analyzed using Cell Quest software. An FSC x FL2 profile was used for establishing an inclusion gate for non-iRBCs. Parasitized RBCs were then quantified by quadrant analysis. Normal RBCs and parasitized RBCs in 15 μg/mL ethylene glycol tetraacetic acid (EGTA) were used as controls. Invasion inhibition was calculated as 100 × (% parasitemia in control −  %parasitemia in test)/(% parasitemia in control).

### Standardizing *Plasmodium berghei* and *Plasmodium yoelii* infection in BALB/c mice

Cryopreserved *P. yoelii* 17XL and *P. berghei* ANKA rodent malarial strains stored in Krebs solution (0.85 % NaCl, 5 % glucose and 4.2 % (p/v) sorbitol) were thawed, heated at 37 °C and subsequently washed with a non-supplemented RPMI medium to produce in vivo malarial infection in BALB/c mice (obtained from the Universidad Nacional de Colombia’s mouse-breeding facility). The obtained strain, having 80 % viability, was centrifuged at 1,500 rpm for 5 min, as previously reported (Ramos-Avila et al. 2007; Spencer-Valero et al. 1998; Lewis-Hughes and Howell 1984; Mons et al. 1983); the pellet containing about 1 × 10^7^ infected red blood cells (iRBCs) was suspended in 1 mL RPMI and immediately used for intraperitoneally inoculating five BALB/c mice, each administered with an average of 2 × 10^6^ iRBCs. Parasitemia in all infected animals was monitored by Wright staining of blood smear, showing that mice became parasitized by the second to sixth day after infection, and that parasitemia levels increased slowly until animals died 10–12 days after having been infected (Ramaiya et al. 1987; Smalley and Butcher 1975).

Given the importance of establishing appropriate in vitro models for testing potential anti-malarial agents against different *Plasmodium* strains, such as those causing rodent malaria, a key step is to maintain a cell culture consisting of some of these agents for periods longer than 10 days. One of the goals of this work was to establish two rodent malarial strains in a controlled cell culture (*Plasmodium berghei* and *Plasmodium yoelii).*

### Antimalarial in vivo activity of antibodies induced by MSA-2 peptide mimetics

Groups of five BALB/c mice were used for testing each antibody sample. Groups were distributed as follows: isotonic saline solution was used for group 1 (control group), clone 6 eluted at 100 mM Ig anti ψ-129 for group 2, clone 7 eluted at 50 mM Ig anti ψ-129 for group 3, clone 7 eluted at 100 mM Ig anti ψ-129 for clone 4, 50 mM Ig anti ψ-131 for clone 5, 100 mM Ig anti ψ-131 for clone 6 and 500 mM Ig anti ψ-131 for clones 7 and 8. A non-relevant polyclonal serum from a mouse immunized with *Papilloma* virus-like particles (VLPs) was used as negative control.

Antibody passive transfer experiments had the following scheme: the first passive transfer was conducted on day −1 by iv administration of 250 μL Igs for each individual. Mice were infected with 50,000 *P. berghei* iRBCs/μL administered in isotonic saline solution on day 0. The second and third passive transfers were made on days 2 and 4 by administering the same immunoglobulin dose. Parasitemia percentage was evaluated from day 6 to 20. A second malaria infection was made on day 21 by ip administration of 100,000 *P. berghei* iRBCs/μL. The 4, 5 and 6th passive transfers were made on days 25, 28 and 30 using similar Ig dose to the starting ones, by iv administration. Percentage parasitemia was assessed from day 25 to day 40.

Immunoglobulin passive transfer experiments and BALB/c mouse challenge with *P. yoelii* were made by forming groups of four to five animals as follows: groups 1 and 10 were injected with isotonic saline solution, group 2 with chloroquine, group 3 with Ig anti ψ-129-6-50 mM, group 4 with Ig anti ψ-129-6-100 mM, group 5 with Ig anti ψ-129-7-50 mM, group 6 with Ig anti ψ-129-7-100 mM, group 7 with 50 mM Ig anti ψ-131, group 8 with 100 mM Ig anti ψ-131 and group 9 with 500 mM Ig anti ψ-131.

Given the stronger virulence of *P. yoelii* compared to the *P. berghei* strain, the immunization scheme was modified as follows: the first passive transfer was carried out on day −1 by administering iv 250 μL Igs to each individual. Mice were iv infected with *P. yoelii* on day 0 by injecting 2,000 iRBCs/μL. The second and third passive transfers were performed on days 2 and 5 using a dose equivalent to the first. Parasitemia percentage was assessed from day 6 to 28. A second malaria infection with *P. yoelii* was carried out on day 13 by ip injection of 5,000 iRBCs/μL. The 4th passive transfer was conducted on day 15, using a similar dose of Ig. The experimental challenge with *P. yoelii* for groups 7, 8, 9 and 10 was conducted following the following scheme. The first passive transfer was carried out on day 0, by iv injecting 100 μL Igs per animal. Experimental infection was made on the same day by iv injecting 2,000 *P. yoelii* iRBCs/μL. Animals were passively transferred on days 1, 2 and 4 with 200 μL Igs per mouse. Parasitemia appearance was assessed from days 3 to 9.

## Results

### Bioinformatics

Blast tool for multiple alignment of protein fragments performed based on the MSA-2^21–40^ or 4044 peptide sequence demonstrated significant high homology and identity percentages with hypothetical, putative and orthologous sequences on *Plasmodium* rodent malaria species such as *P. berghei* and *P. yoelii*.

Since so far MSA-2 has no reported orthologous sequences in the *P. yoelii* genome, using the peptide 4044 amino acid sequence as the basis to be analyzed against all proteins reported for *P. yoelii* and *P. berghei*, an important number of hypothetical, putative and expressed protein-genes were found, 56 and 44, respectively, all of them located in different cell compartments such as nuclei, cytosol and in specific organelles such as rhoptries. Some representative examples from the whole obtained data show that on comparing the *Plasmodium falciparum* MSA-2^21–40^ amino acid sequence, a fragment from ^22^N to I^39^ has a 67 % identity and 78 % homology with a *Plasmodium berghei* strain ANKA transcription initiation TFIID-like coded PB000459. Similarly from the same *P. falciparum* MSA-2, the fragment ^22^N to I^39^ has a 61 % identity and 72 % homology with a TATA element modulatory factor of *Plasmodium yoelii* strain 17XL. Also, a 17-residue fragment from ^22^N to M^37^ on the *P. falciparum* MSA-2^21–40^ peptide has an 80 % identity and 80 % homology with a *Plasmodium yoelii* hypothetical protein PY06720 and 64 % identity and 71 % homology for a *P. falciparum* MSA-2^21–40^ with a 16-residue fragment from ^23^E to Y^35^ located in the hypothetical protein PY05325 of *Plasmodium yoelii* strain 17XL. On the other hand, the 15-residue fragment from the *P. falciparum* MSA-2^21–40^ peptide has a 64 % identity and 71 % homology with a fragment located on the putative protein coded PB500001 of *Plasmodium berghei* strain ANKA.

Remarkably, a 15-residue peptide from ^22^N to Y^35^ from the *P. falciparum* MSA-2^21–40^ was identified in the erythrocyte membrane protein 3 PFEMP3, PY02048 (gene bank EAA21466.1) of *Plasmodium yoelii yoelii* 17XNL having 64 % of identity and 64 % homology and an 18-residue fragment from ^21^K to S^38^ of the *P. falciparum* MSA-2^21–40^ peptide was identified in the putative yir2 antigenic protein of *Plasmodium yoelii yoelii* having 56 % identity and 56 % homology as well as in the putative yir1 protein of *P. yoelii yoelii* peptide fragment from ^21^K to Y^35^ having 60 % identity and 67 % homology; other gene identified as sharing a 15-peptide sequence was the putative yir4 protein gene bank codes EAA22494.1 and EAA22744.1 having 60 and 53 % of identity, respectively. Also a 19-residue fragment of the *P. falciparum* MSA-2^21–40^ peptide was identified in the maebl hypothetical protein of *Plasmodium yoelii yoelii* 17XNL as PY03552, gene bank code EAA15281.1. Among several examples, a 16-peptide fragment of the N-terminal MSA-2 *P. falciparum* antigen, a 16-residue peptide from ^26^Y to N^36^ was identified in the vesicle transport protein of *Plasmodium berghei* strain ANKA genome coded PB000004, EMB code CAH93534.1, having 73 % identity and 82 % homology as well as a 17-residue peptide from ^28^N to M^37^ was found in the glycoxalate II family protein, putative of *Plasmodium berghei* strain ANKA coded as PB000214.01 and EBM: CAH95663.1, having 67 % identity and 67 % homology. A 19-residue fragment in the same region has a 46 % identity with the 235 kDa rhoptry protein EAA16521.1, PY04630 of *P. yoelii yoelii* and 53 % identity with the reticulocyte binding protein CAI02683.1 and 73 % identity with the putative *N*-acetyltransferase EMB code CAI04236.1, PB000192.01 of *Plasmodium berghei* in a 19-residue peptide from ^30^F to I^39^. Other peptide homologs were found in the protein phosphatase EAA21747.1 of P. yoelii with 78 % identity as well as 43 % with the mechanoselective ion channel putaitive protein EAA17950.1, PY05855 of *P. yoelii*.

### Designing, synthesis and structural characterization of high binding MSA-2-derived peptide 4044 and its amide-reduced peptide mimetics

**Fig. 1:**
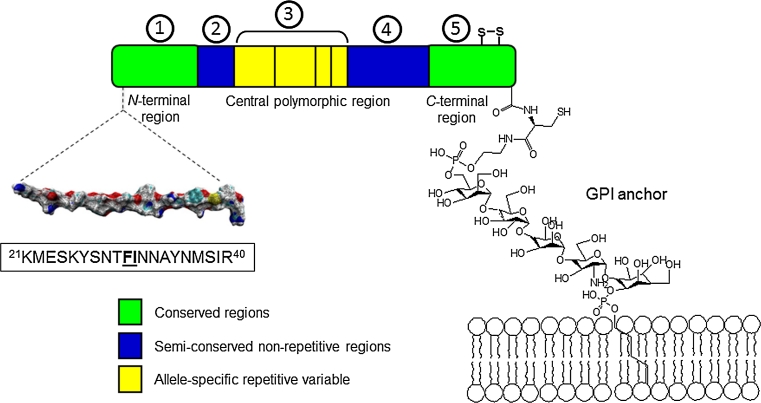
Schematic structure of the merozoite surface antigen-2. MSP-2 gene block organization is represented by different colors in regions and denoted by numbers from 1 to 5, highlighting the 4044 peptide NMR-solution structure located at the molecule N-terminal. Critical binding residues are underlined. MSA-2 is anchored to the parasite surface by a GPI motif

**Fig. 2:**
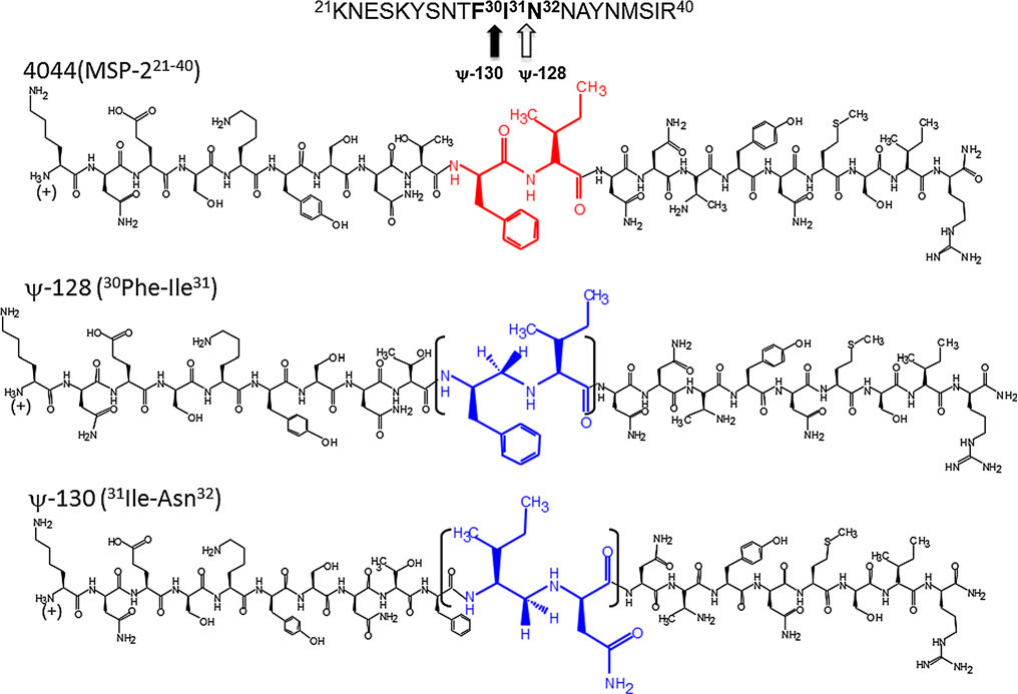
Experimental design for the site-directed synthesis of pseudopeptides ψ-128 and ψ-130. The amide type peptide bond [CO–NH] was substituted for its reduced amide isostere bond ψ–[CH_2_NH] between residues ^30^Phe-Ile^31^ in analog ψ-130, and between residues ^31^Ile-Asn^32^ to produce analog ψ-128

**Fig. 3:**
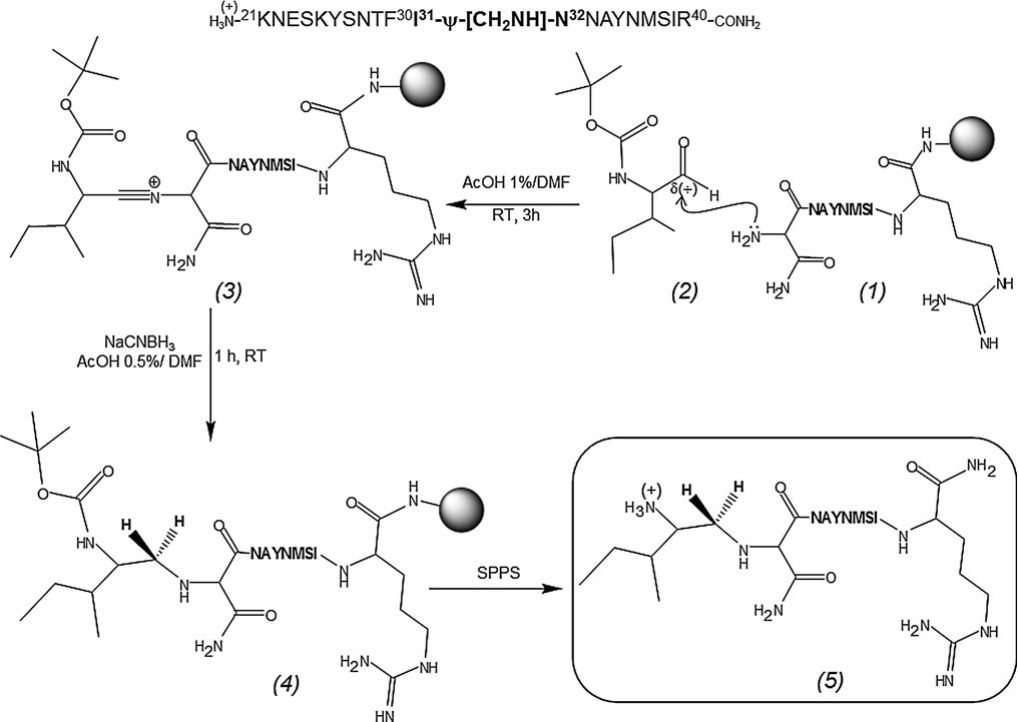
Solid-phase synthesis of malaria MSA-2 antigen-derived peptide mimetics. (*1*) The amino primary function of Asn residue in the sequence anchored to the methylbenzhydrylamine resin (MBHA) was condensed with the carbonyl carbon of synthon* t*-Boc-***L***–Ile–H (*2*), thereby forming the iminium ion in the product (*3*). The formation of this ion led to a methylene group formation in product (*4*) after being treated with the NaCNBH_3_ reducing agent. After incorporating the remaining normal amino acids into the peptide chain, the final expected product, ψ-130 (*5*), was generated when it was cleaved from the resin by treatment with low and high HF concentrations. *SPPS* was used for solid-phase peptide synthesis

As mentioned, peptide coded 4044 (MSA-2^21−40^) is located in conserved block 1 of the conserved amino-terminal region in the MSA-2 antigen sequence as observed in Fig. 1. This protein portion is unstructured as determined by ^1^H-NMR experiments conducted in solution. The RBC binding motif, defined by the ^30^F–I–N^32^ residues, served as the basis for designing a single peptide bond site-directed substitution by the ψ–[CH_2_–NH] isostere bond, producing ψ-128 (F–I) and ψ-130 (I–N) pseudopeptides and their corresponding polymeric forms, ψ-129 and ψ-131, respectively as shown in Fig. 2. The synthetic transformation of *N*-α-amino protected phenylalanine *t*-Boc-L–Phe–OH to a required *t*-Boc-L–Phe–H amino aldehyde form as the building block to be further incorporated into a growing peptide resin-defined sequence is displayed in Fig. 3. This two-stage strategy includes derivatizing compound (*1*) to its carboxamide form to protect the amino acid carbonyl carbon which is treated later with a strong reducing agent, aluminum and lithium hydride, to form a five-member partial ring including lithium, which is then hydrolyzed to generate the amino acid aldehyde form (*2*). Compound (*3*) harboring the *iminium* ion is susceptible to a specific reduction with sodium cyanoborohydride (NaCNBH_3_), to allow (*4*). The process has an overall 60–70 % yield represented by compound (*5*) when carried out with any given amino acid, except asparagine and glutamine (Asn and Gln) which need to be treated in a different way, as discussed elsewhere (Carreño et al. 2011).

### Spectroscopic characterization of *t*-Boc–Phe and* t*-Boc–Ile amino acids and their derivatives

#### *t*-Boc-***L***–Phe derivatives

***t*** **-** ***Boc*** **-** ***L*** **–** ***Phe*** **–** ***OH***, Rf = 0.40 ethyl acetate:*n*-hexane (1:2).

^1^H-NMR (dimethylsulfoxide-D6 (D, 99.9 %)): δ = 1.42 ppm (9H, C(CH3); δ = 2.66 ppm (2H, Hβ); δ = 5,76 ppm (1H, Hα); δ = 7,17 ppm (2H, H2-6-aromatic ring); δ = 7,22 ppm (2H, H3-5-ring) δ = 7,23 ppm (2H, H4-aromatic ring); δ = 7,28 ppm (1H, NHα); δ = 2.5 ppm (m, 6H, dimethyl sulfoxide).

FT-IR (amorphous solid): ν(cm^−1^) = 3,299.14, 3,246.12, 2,974.23, 1,707.43, 1,644.67, 1,496.54, 1,405.60, 1,359.34, 1,272.23, 1,156.45, 774.09.

***t*** **-** ***Boc*** **-** ***L*** **–** ***Phe*** **–** ***N(CH***_***3***_ ***)OCH***_***3***_, Rf = 0.50 ethyl acetate:*n*-hexane (1:2).

^1^H-NMR (dimethyl sulfoxide-D6 (D, 99.9 %)): δ = 1.39 ppm (9H, C(CH3); δ = 2.81 ppm (2H, Hβ); δ = 3,18 ppm (3H, N–(CH3)); δ = 3,65 ppm (3H, N–O–(CH3)); δ = 5,25 ppm (1H, Hα); δ = 7,19 ppm (2H, H2-6-aromatic ring); δ = 7,24 ppm (2H, H3-5-aromatic ring) δ = 7,25 ppm (2H, H4-aromatic ring); δ  = 7,28 ppm (1H, NHα); δ = 2.5 ppm (m, 6H, dimethyl sulfoxide).

FT-IR (oiled film): ν(cm^−1^) = 3,299.14, 3,246.12, 2,974.23, 1,894.98, 1,657.14, 1,644.67, 1,496.54, 1,405.60, 1,359.34, 1,272.23, 1,156.45, 774.09.

***t*** **-** ***Boc*** **-** ***L*** **–** ***Phe*** **–** ***H***, Rf = 0.69 ethyl acetate:*n*-hexane (1:2).

^1^H-NMR (dimethyl sulfoxide-D6 (D, 99.9 %)): δ = 1.48 ppm (9H, C(CH3); δ = 2.81 ppm (2H, Hβ); δ = 5,01 ppm (1H, Hα); δ = 7,17 ppm (2H, H2-6-aromatic ring); δ = 7,23 ppm (2H, H3-5-aromatic ring) δ = 7,25 ppm (2H, H4-aromatic ring); δ  = 7,32 ppm (1H, NHα); δ = 9,63 ppm (1H, formyl); δ = 2.5 ppm (m, 6H, dimethyl sulfoxide).

FT-IR (oiled film): ν(cm^−1^) = 3,360.43, 3,246.12, 2,974.23, 1,677.05, 1,644.67, 1,496.54, 1,405.60, 1,359.34, 1,272.23, 1,156.45, 774.09.

#### *t*-Boc-***L***–Ile derivatives

***t*** **-** ***Boc*** **-** ***L*** **–** ***Ile*** **–** ***OH***, Rf = 0.66 ethyl acetate:*n*-hexane (1:1).

^1^H-NMR (dimethyl sulfoxide-D6 (D, 99.9 %)): δ = 0,94 ppm (3H, Hγ); δ = 0,97 ppm (3H, Hδ); δ = 1,20 ppm (2H, Hγ); δ = 1.45 ppm (9H, C(CH3)); δ = 1,92 ppm (1H, Hβ); δ = 4,29 ppm (1H, Hα); δ = 7,25 ppm (1H, NHα); δ = 2.5 ppm (m, 6H, dimethyl sulfoxide).

FT-IR (amorphous solid): ν(cm^−1^): 3,352.65, 3,299.24, 2,963.19, 1,726.67, 1,699.54, 1,673.08, 1,530.43, 1,451.60, 1,412.79, 1,299.21, 1,160.34, 1,016.01, 776.44.

***t*** **-** ***Boc*** **-** ***L*** **–** ***Ile*** **–** ***N(CH***_***3***_ ***)OCH***_***3***_, Rf = 0.54 ethyl acetate:*n*-hexane (1:1).

^1^H-NMR (dimethyl sulfoxide-D6 (D, 99.9 %)): δ = 0,89 ppm (3H, Hγ); δ = 0,93 ppm (3H, Hδ); δ = 1,12 ppm (2H, Hγ); δ = 1.43 ppm (9H, C(CH3)); δ = 2,80 ppm (1H, Hβ); δ = 3,21 ppm (3H, N–(CH3)); δ = 3,78 ppm (3H, N–O–(CH3)) δ = 4,61 ppm (1H, Hα); δ = 7,25 ppm (1H, NHα); δ = 2.5 ppm (m, 6H, dimethyl sulfoxide).

FT-IR (powdered): ν(cm^−1^): 3,352.65, 3,299.24, 2,963.19, 1,699.54, 1,688.14, 1,654.77, 1,530.43, 1,451.60, 1,412.79, 1,299.21, 1,160.34, 1,016.01, 776.44.

***t*** **-** ***Boc*** **-** ***L*** **–** ***Ile*** **–** ***H***, Rf = 0.58 ethyl acetate:*n*-hexane (1:1).

^1^H-NMR (dimethyl sulfoxide-D6 (D, 99.9 %)): δ = 0,91 ppm (3H, Hγ); δ = 0,94 ppm (3H, Hδ); δ = 0,96 ppm (2H, Hγ); δ = 1.45 ppm (9H, C(CH3)); δ = 2,03 ppm (1H, Hβ); δ = 4,37 ppm (1H, Hα); δ = 7,25 ppm (1H, NHα); δ = 9,66 ppm (1H, formyl); δ = 2.5 ppm (m, 6H, dimethyl sulfoxide).

FT-IR (oiled film): ν(cm^−1^): 2,963.21, 1,693.09, 1,530.43, 1,451.60, 1,412.79, 1,299.21, 1,160.34, 1,016.01, 776.44.

**Fig. 4:**
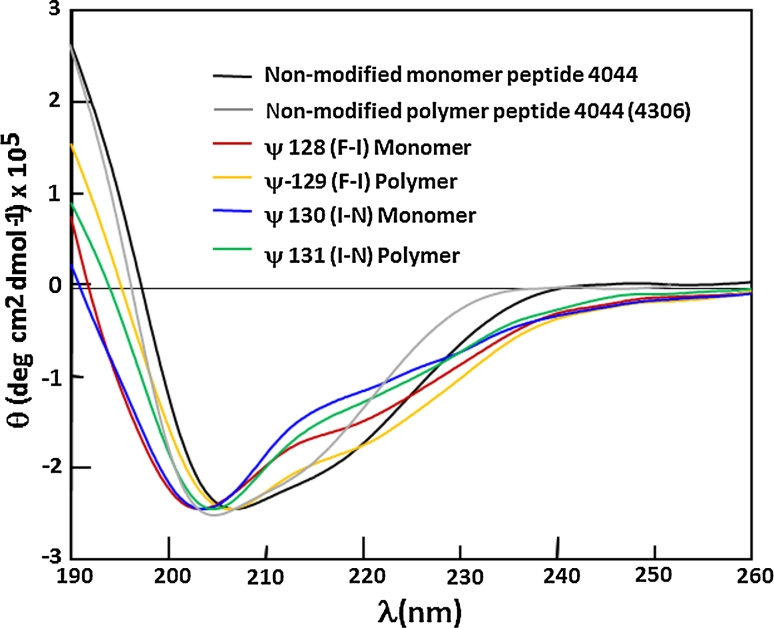
Secondary structure profiles for all 4044-derived peptide mimetics by circular dichroism (CD). In all cases the non-modified peptide 4044 and its peptide mimetics were analyzed as both monomer and polymer forms. Molar ellipticity is denoted by Θ whose units are deg × cm^2^ × dmol^−1^

**Fig. 5:**
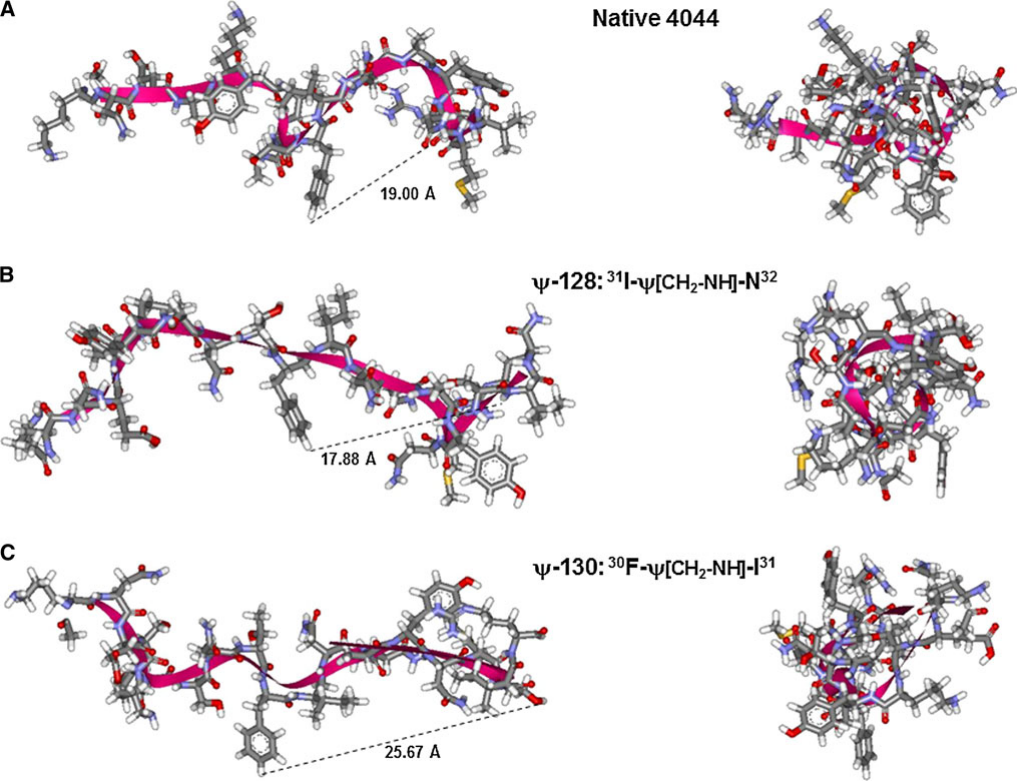
The effect of introducing a single ψ–[CH_2_NH] peptide bond modification on the MSA-2^21–40^ fragment backbone and its relevance to the molecule 3D structure. A consensus structure for peptide 4044 and its pseudopeptides ψ-128 and ψ-130, all having the lowest total energy are displayed in ball and sticks representing the molecular 3D structure of target molecules based on multidimensional ^1^H-NMR experiments. The molecule backbone is shown by red ribbons. Conformation properties for peptide MSA-2^21−40^ (peptide 4044) in panel (**a**) and its analog pseudopeptides ψ-128 in (**b**) and ψ-130 in (**c**). *Left panels* show all molecules’ side view and *right panels* show the molecules’ front view from the N- to C-terminus. Distance between ^30^F and ^38^S is shown in Å. *Left panels* show all molecules’ side view and *right panels* show the molecules’ front view from N- to C-terminus

Subsequently, the synthon (*3*) was incorporated into the in situ growing peptide chain during synthetic solid phase to be submitted to a second reduction in gentler conditions using sodium cyanoborohydride (NaCNBH_3_), thereby producing target peptide mimetics (as shown in Fig. 3). As mentioned above, the physicochemical characteristics of the molecules produced for this study can be observed in Table 1, defined as native peptide 4044 and its pseudopeptide analogs in their monomeric and polymeric forms. All these molecules had the proper molecular qualities required for carrying out immunological assays and in all cases overall yields were higher than 70 %. When necessary, molecules were purified by RP-HPLC allowing purity higher than 98 %. This new group of molecules was then characterized in terms of the presence of secondary structure elements using circular dichroism (CD) as displayed in Fig. 4. In a 30 % TFE solution, 4044 peptide and its pseudopeptide analogs in both monomer and polymeric forms exhibited α-helical features as judged by the presence of a maximal ellipticity at 190 nm and two minimal ellipticity points at 208 and 222 nm, typical for this type of secondary element, However, these molecules also had more extended structural element features, such as β-strands, highlighting the presence of a single minimum at 218 nm and unstructured regions. These molecules’ structural preference was thus intermediate, ranging from a distorted helix to an unstable β-strand or can be randomly organized. To confirm solution structure properties of this molecular family, ^1^H-NMR experiments were conducted to obtain representative molecular models as shown in Fig. 5 that will be discussed below.

### Molecular structure calculation

In all cases, a set of 50 structures was obtained from the native 4044 peptide’s coordinates as well as for its pseudopeptides ψ-128 and ψ-130, satisfying experimental constraints when 131 data NOEs-derived from distance restraints which were previously classified according to signal-strength. Then dihedral angles restraints were added in molecular dynamics calculations for ensuring accuracy. As expected due to a low number of medium-strength NOE, signals for both peptide mimetics display a high degree of flexibility in the whole pseudopeptide backbone. The obtained molecular structures showed lower total energy values.

Given the data’s relevance, ^1^H-NMR studies were then made by applying two-dimensional techniques such as nuclear Overhauser effect spectroscopy (NOESY) to generate more accurate data regarding the three molecules’ 3D structure. 3D molecular models based on NMR data were thus obtained for each molecule. The molecular structures showed the stability of features displaying α-helix characteristics; such features were seen to be stronger in native peptide 4044 than in either peptidomimetic ψ-128 or ψ-130. This was seen by the presence of several hydrogen bonds which are usually displayed by helical structures. The more relaxed structural trend in the two peptide mimetics was also evident. The structural impact of the two new degrees of freedom in both molecular analogs was dramatic, in such a way that some characteristic features from α-helices displayed by the native peptide became lost in the new analogs, displaying more relaxed conformations. Figure 5 displays the most representative molecular models for 4044 peptide and its ψ-128 and ψ-130 pseudopeptide analogs. Modification of specific peptide bonds allowed new molecular constraints in the global molecular structure which, as will be discussed, will have a dramatic impact on their immunological activity.

### Hybridoma cloning and antibody isotype switching and reactivity

**Fig. 6:**
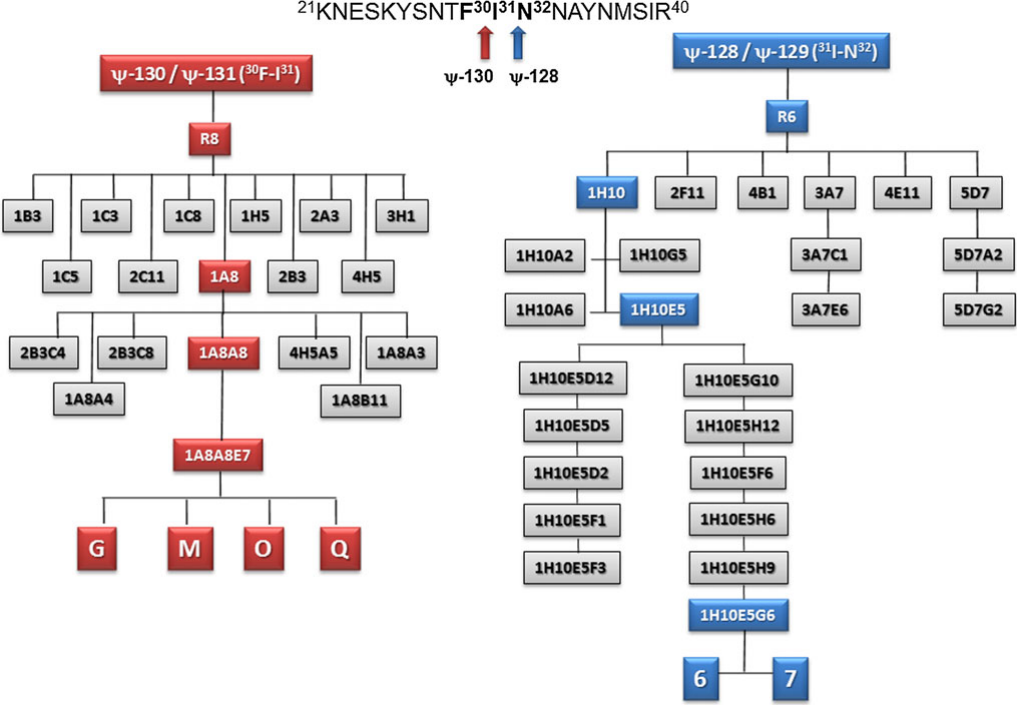
Genealogical tree of antibody clones induced by pseudopeptides ψ-128 and ψ-130 derived from the *Plasmodium falciparum* MSA-2 antigen so-called HABP 4044. Each level in the scale represents a sub-cloning of new antibody populations

**Fig. 7:**
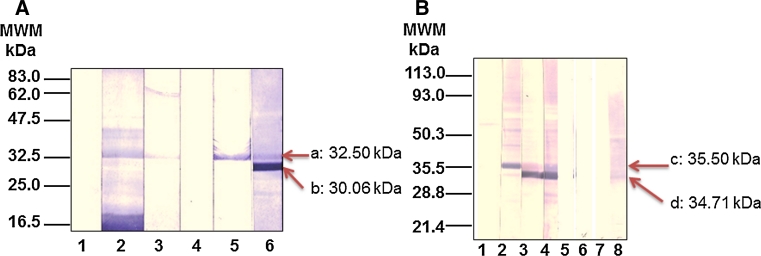
Reactivity of *Aotus* polyclonal antibodies and hybridoma-IgM antibodies to MSA-2 peptide mimetics. **a** Antibody reactivity of hybridoma-IgM produced antibodies induced by MSA-2 peptide mimetics against a recombinant MSA-2 expressed in *E. coli*. *Lane 1* for the negative control, *lane 2* shows the reactivity of an anti-His antibody, *lanes 3* and 4 show the reactivity of IgM induced by ψ-130 and *lanes 5* and *6* the reactivity of IgM induced by ψ-128. **b** Antibody reactivity of *Aotus* monkeys immunized with MSA-2 peptide mimetics against a recombinant MSA-2 expressed in *E. coli*; *lane 1* the negative control, *lane 2* the reactivity of an anti-His antibody, *lane 3* the reactivity of polyclonal antibodies induced by ψ-130 in *Aotus* monkeys, *lane 4* the reactivity of polyclonal antibodies induced by ψ-128 in *Aotus* monkeys, *lanes 5* and 6 display the reactivity of pre-immune sera from monkeys and *lanes 7* and 8 sera reactivity of pre-immune and post third dose samples of monkeys immunized with native 4044 peptide

Stable and highly specific antibodies directed against MSA-2 were obtained after several steps involving limited dilution cloning and sub-cloning of parent hybridomas induced by both ψ-128 (^31^I–N^32^) and ψ-130 (^30^F–I^31^) peptide mimetics, as described by the family tree displayed in Fig. 6, left panel. Multiple cloning led to obtaining homogeneous monoclonal antibodies expressing cells which were letter-coded G, M, O and Q for those induced by ψ-130 peptidomimetic and number-coded 6 and 7 for those induced by the ψ-128 peptide mimetic (Fig. 6, right panel). A group of hybridomas was generated; at first these presented the IgM isotype. This pentavalent molecule served as the basis for carrying out in vitro immunization assays to induce isotype switching to simpler, functional, antibody forms. Table 2 shows the main results obtained by this procedure and its versatility in inducing the drastic change of IgM isotype to IgG1, IgG2a, IgG2b and IgG3 whose malaria infection neutralizing activity was evident as will be discussed below. Given the importance of this family of antibodies, Western blot experiments allowed to analyze their cross-reactivity against an N-terminal MSA-2 recombinant fragment (rMSA-2). As observed in Fig. 7, reactive clones induced by ψ-128 and ψ-130 revealed two strong specific bands having relative molecular weights ranging between 30.06 and 35.50 kDa, the slower being recognized by an anti-histidine antibody. The rMSA-2 expressed fragment in an *E. coli* membrane lysate was resolved on SDS-PAGE 10 % polyacrylamide gels and electro-transferred to a nitrocellulose paper as discussed in “Materials and methods”.

**Table 2:** Isotype switching of clones induced by the ψ-128 and ψ-130 pseudopeptides derived from the MSA-2^21–40^ peptide

Monomer	Polymer	Clon	Original Ig isotype in the parent cell clone					In vitro isotype switching in isolated immunoglobulins					
			IgG1 %	IgG2a %	IgG2b %	IgG3 %	IgM %	Eluted fractions (ionic strength) mM	IgG1 %	IgG2a %	IgG2b %	IgG3 %	IgM %
ψ-128	ψ-129	6	0.00	0.00	0.00	5.70	94.30	50	6.00	13.80	11.86	62.53	5.78
								100	3.59	12.57	11.93	65.04	6.82
								500	4.10	15.30	8.39	61.30	10.85
		7	0.00	0.00	2.95	40.60	56.30	50	16.08	14.31	42.87	10.42	16.31
								100	17.28	17.87	36.93	13.15	14.77
								500	20.22	22.45	24.30	15.40	17.62
ψ-130	ψ-131	G	1.70	0.23	0.59	3.52	97.65	50	8.02	5.96	3.91	0.28	81.84
		M	1.25	0.00	0.33	1.95	96.50						
		O	0.16	0.00	0.23	1.88	97.70	100	6.08	9.23	7.45	0.025	77.22
		Q	0.00	0.00	0.18	5.30	94.50	500	3.52	2.39	1.81	0.00	92.28

**Fig. 8:**
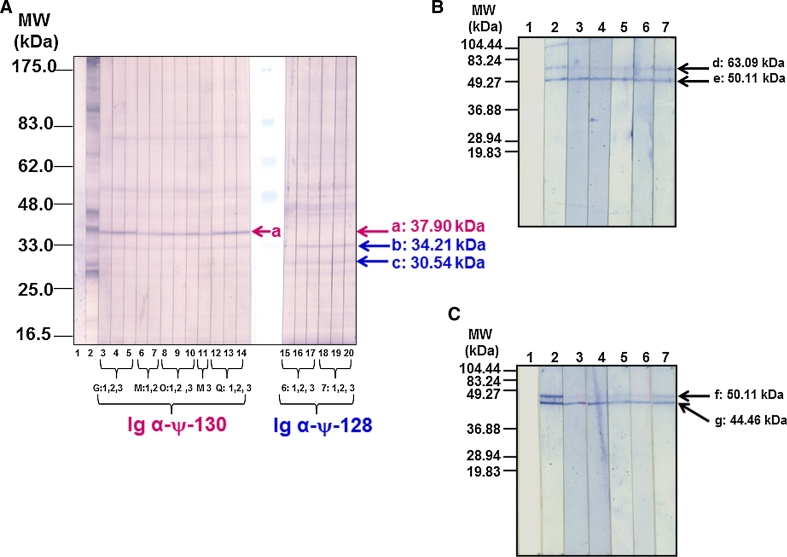
Reactivity of IgG monoclonal antibodies generated by the peptide mimetics ψ-128 and ψ-130 derived from native peptide 4044 by Western blot analysis. **A** The reactivity of monoclonal antibodies induced by pseudopeptide ψ-130 can be observed at the left side of the gel by the recognition of band (*a*) about a 37.9 kDa of relative molecular weight and the reactivity of ψ-128-induced monoclonal antibodies can be observed on the right by recognition of bands (*b*) and (*c*) having relative molecular weights of 30.54 and 34.21 kDa, respectively.** B** A cytosol protein extract obtained from *Plasmodium berghei* was resolved by SDS-PAGE and electro-transferred to nitrocellulose for analyzing the monoclonal antibody reactivity.** C** A cytosol protein extract from *Plasmodium yoelii* was obtained as in the previous case. In A a *Plasmodium falciparum* lysate (3 mg) electrophoresis was carried out on an acrylamide–bisacrylamide gel having 7.5–15 % gradient at 100 v for 4 h and then electro-transferred to nitrocellulose paper and then cut into 0.5 cm strips. Each strip was incubated with the corresponding antibody or as part of the control group. In **b** and **c**, *lane 1* is the negative control (i.e. blocking solution), *lanes 2* and 3 display the reactivity of monoclonal antibodies coded 6 and 7 induced by the pseudopeptide ψ-128 (^31^I–N^32^) and lanes 4–7 display the reactivity of monoclonal antibodies coded G, M, O and Q induced by the pseudopeptide ψ-130 (^30^F–I^31^)

After successful clone and isotype characterization, monoclonal antibodies induced by site-directed designed peptide mimetics were tested for their reactivity against native MSA-2 antigen from a *Plasmodium falciparum* lysate resolved on a 7.5–15 % gradient gel by SDS-polyacrylamide gel electrophoresis. Western blot analysis for specific antibody reactivity (Fig. 8) displayed reactivity profiles for both sets of mAbs αψ-128 and αψ-130 antibodies. The versatility of modifying adjacent residues (involving two different residues, such as ^30^Phe-Ile^31^ and ^31^Ile-Asn^32^) induced two groups of antibodies having strong, differential, specific reactivity; the first group induced by ψ-130 (^30^Phe-Ile^31^) recognized a 37.90 kDa band having relative mobility and the second group of antibodies induced by ψ-128 (^31^Ile-Asn^32^) specifically recognized two bands having 34.21 kDa and 30.54 kDa relative molecular weights as displayed in Fig. 8a. An important experiment allowed evidencing for the first time the existence of a possible immature construct expressed on the cytosol compartment of two rodent malaria species such as *Plasmodium berghei* and *Plasmodium yoelii* strains, as can be observed in Fig. 8b and c. In the cytosol of *P. berghei* two strong bands were stained with monoclonal antibodies to the 4044-reduced pseudopeptides, a slow band at 63.09 kDa and a faster one at 50.11 kDa. Similarly in cytosol of *P. yoelii* two strong bands were also stained with monoclonal antibodies to the 4044-reduced pseudopeptides, a slow band at 50.11 kDa and a faster one at 44.46 kDa. In both cases, antibodies induced by the ψ-128 exhibited a strong reactivity for the band having higher relative molecular weight.

### Mapping the MSA-2^21–40^ epitope

The MSA-2^21–40^ N-terminal-conserved sequence represented by peptide 4044 (^21^KNESKYSNTFINNAYNMSIR^40^) possesses important functional features as described by different authors. Firstly, the amino acid sequence ^27^SNTFINNA^34^ reported by Jones (Jones et al. 1996) represents an N-terminal epitope; secondly, this protein fragment possesses a central high binding motif to RBCs represented by the ^30^FIN^32^ amino acid sequence reported by Ocampo et al. (2000); thirdly Patarroyo et al. (2011) have recently reported a binding register to pockets 1–9 of the HLA-DRβ1 constituted by ^30^FINNAYNMS^38^ amino acid sequence, which after being strategically modified by replacing specific amino acids with others having physical and chemical characteristics turned it into a protective immunogen that is considered as a component for a sub-unit based malaria vaccine. However, the native 4044 molecule has proven to be poorly immunogenic and non-protective against malaria when administered as a non-modified sequence. Our experiments have demonstrated that isostere bonds strategically located at specific places within peptide sequences can be introduced as novel important molecular elements for obtaining functional immunogens as discussed below.

**Fig. 9:**
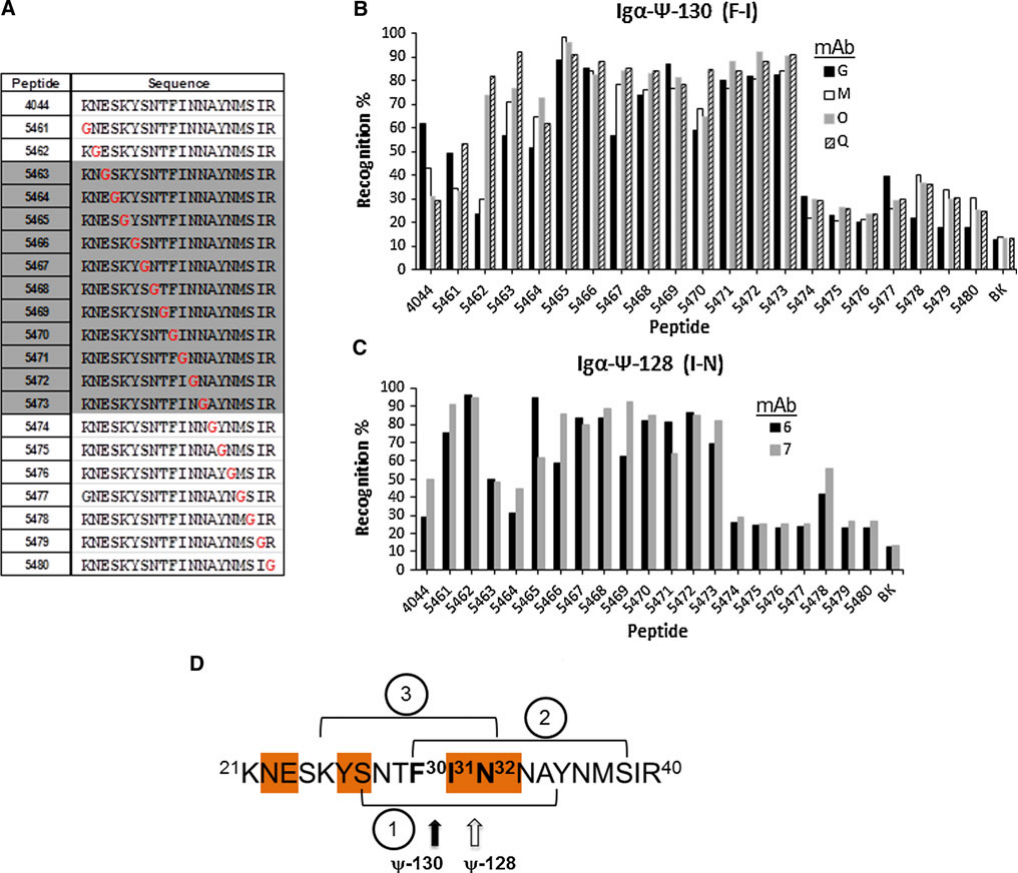
Mapping the N-terminal MSA-2 epitope. The peptide 4044 glycine scan analog peptides is displayed in **a**, **b**. The reactivity pattern of monoclonal antibodies coded as G, M, O and Q induced by the pseudopeptide ψ-130 (^30^F–I^31^) against the 4044 peptide glycine scan. **c**. The reactivity pattern of monoclonal antibodies coded 6 and 7 induced by the pseudopeptide ψ-128 (^31^I–N^32^) against the 4044 peptide glycine scan. Each bar represents experiments performed in duplicate by ELISA. **d** Schematic representation of the MSA-2^21–40^ N-terminal region in which a redefined epitope is shown in (*3*), which overlap the previously reported (*1*) (Jones et al. 1996) and a described HLA-DRβ1 reading frame motif (*2*) (Patarroyo et al. 2011)

To precisely define the epitope present in the same MSA-2^21–40^ protein fragment, we propose using monoclonal antibodies induced by the reduced amide pseudopeptides each containing a non-natural peptide-bond isostere, one located between the ^30^F–I^31^ amino acid pair on the ψ-130 and the second between the ^31^I–N^32^ on the ψ-128 analog as molecular tools for mapping this protein fragment epitope. The immunochemical tests performed evaluating these mAbs cross-reactivity against a set of glycine scan 4044 peptide analogs are shown in Fig. 9a. As a result, panels b and c of Fig. 9 display a recognition preference of the central portion of the 4044 amino acid sequence. This fact can be explained as: the antibody inducer pseudopeptides had included two new freedom degrees to the entire molecule when the isostere –CH_2_–NH– was incorporated as a –CO–NH– substituent. The effect on the molecule was allowed by introducing two hydrogen atoms replacing an oxygen atom in a given peptide bond. Thus, a replacement of any amino acid in the central part of the molecule with a glycine residue would resemble the two new freedom degrees that both mAbs know how to recognize; in consequence, a new epitope was revealed as the sequence ^25^KYSNTFIN^32^ at the N-terminal of the MSA-2 surface antigen as can be observed in Fig. 9d.

### Establishing in vitro and in vivo infection models for rodent malaria

Bearing in mind the reactivity of this family of monoclonal antibodies, we decided to perform in vivo experiments for evaluating their possible functional activity in two infected rodent malaria models. The characteristic morphology of BALB/c mice erythrocytes infected with *Plasmodium yoelii* 17XL and *Plasmodium berghei* ANKA rodent malaria strains allowed controlling and characterizing these two entities’ erythrocyte cycle. Thus, different malarial parasite blood stages, such as rings, trophozoites and mature schizonts were produced in the blood stages of these *Plasmodium* strains. Each cell culture was maintained under controlled conditions in stages lasting a month for both cases. Preliminary studies for assessing in vitro anti-plasmodium activity were conducted in controlled cultures and showed a defined isotype antibody effect, which was very similar to that observed in the in vivo antimalarial activity assays. In all cases, parasitemia percentages were lower than those in the control experiment, which was normal infection of RBC parasitized with *P. berghei* after two experimental challenges. The parasitemia peak observed toward day 10 was due to the second in vitro challenge. The *P. yoelii* strain was less virulent in in vivo assays (as will be discussed further on). All groups administered with antibodies had better control of parasitemia levels, except for the group treated with EGTA.

**Fig. 10:**
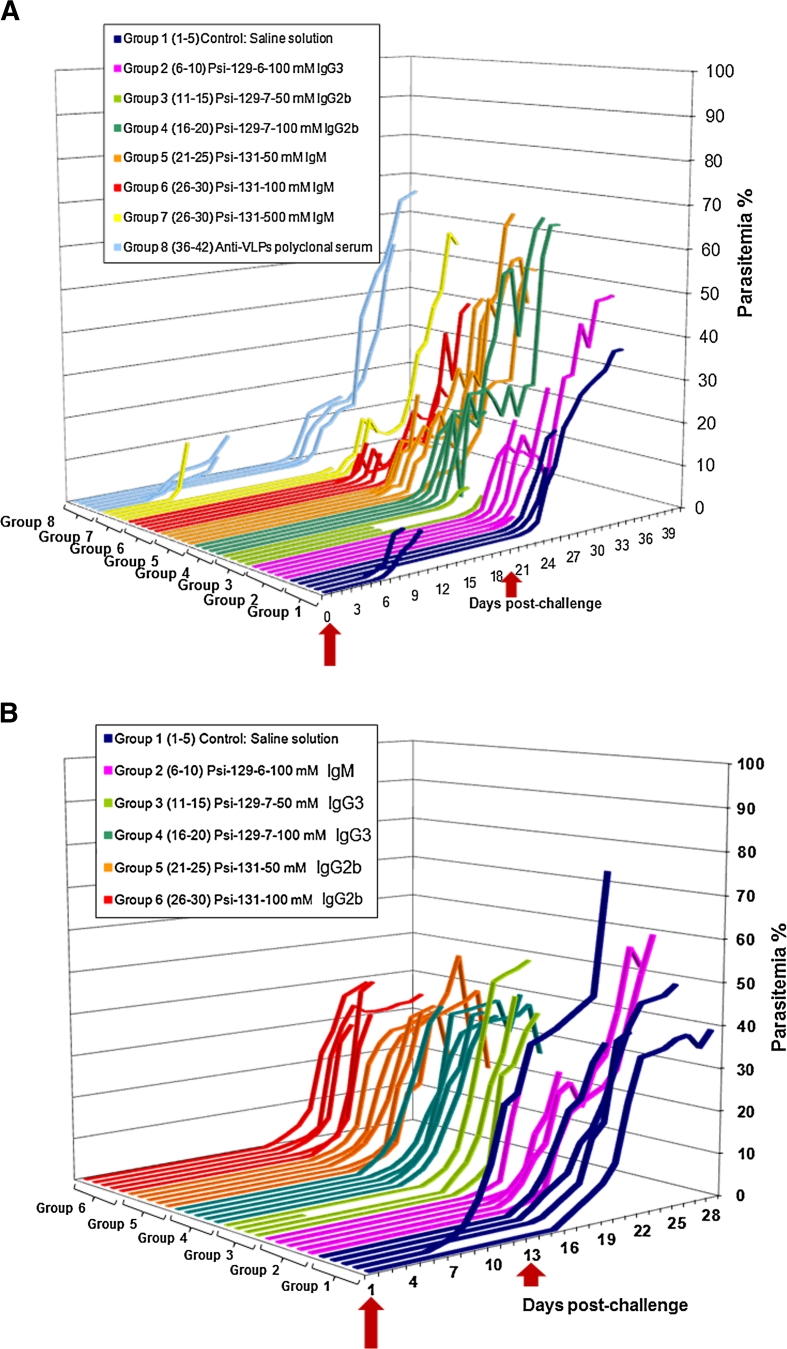
Parasitemia patterns for BALB/c mice passively transferred with pseudopeptide ψ -128 and ψ -130-induced isotype-defined immunoglobulins. **a** *P. berghei*-infected BALB/c mice. **b** *P.-yoelii* infected BALB/c mice. *Arrow*s display time mice were challenged with rodent malaria strains

Once the infection models (in both in vitro controlled cultures and in vivo BALB/c female mice) had been established, the functional activity of monoclonal antibodies induced by peptide mimetics derived from the native peptide 4044 sequence was evaluated by using a designed antibody passive administration scheme for *P. berghei*- and *P. yoelii*-infected BALB/c mice. Thus, passive transference experiments were followed up over a period of 40 and 30 days, respectively, as shown in Fig. 10.

In all cases, the control groups (given (iv) isotonic saline solution) presented higher infection kinetics than those displayed by the study groups which were given pure immunoglobulins having a defined isotype in the same way. These groups also showed a delay in the emergence of significant percentages of parasitemia and were able to manage much higher parasitemia levels, showing that the antibodies were having a modulating effect on the parasite. Remarkably, the groups of animals which were given isotonic saline solution became infected faster after experimental challenge than the study groups, managing lower parasitemia levels which were enough to cause the death of the animals (this was not happening in the study groups).

**Fig. 11:**
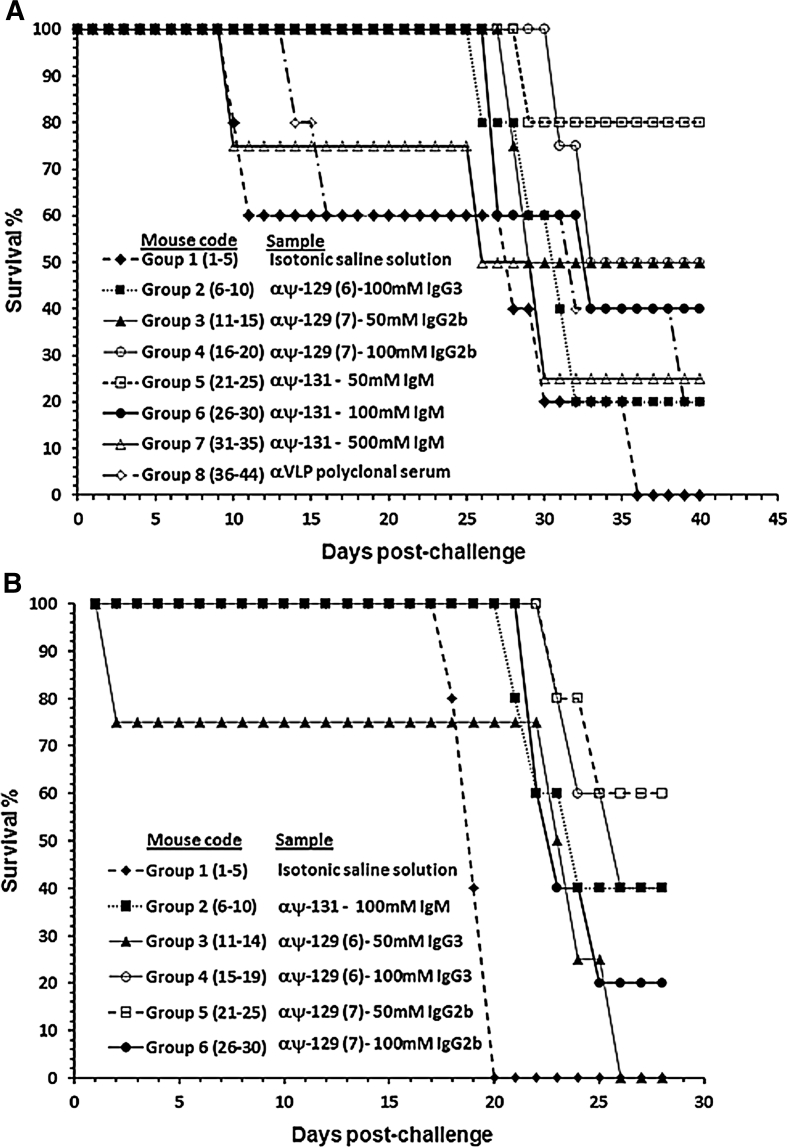
Survival profiles for BALB/c mice passively transferred with pseudopeptide ψ-128 and ψ-130-induced isotype-defined immunoglobulins. Experimental challenge was made with **a** *Plasmodium berghei* and **b** *Plasmodium yoelii*. In both cases animals were distributed in groups of five individuals as shown in parenthesis. Group 1 was administered with isotonic saline solution as being the experimental group. In A, test animal groups were administered with purified monoclonal antibodies induced by the pseudopeptides ψ-128/129 (I^31^–N^32^) and ψ-130/131 (^30^F–I^31^); in B animals were administered with purified monoclonal antibodies induced by the pseudopeptide ψ-128/129 (I^31^–N^32^). Survival profiles reveal the immunotherapeutical effect of passively transferring pure immunoglobulins into rodent malaria-infected BALB/c mice with lethal doses of *Plasmodium*. All animals administered with isotonic saline solution died during the experiment and numbers in parentheses represent mice codes

The animals from the second group which were passively transferred with antibodies induced by ψ-128 (ψ-129) managed to significantly control infection, reversing the *P. berghei* strain’s infectious effect. Animals from groups 6 and 7, passively transferred with antibodies induced by ψ-130 (ψ-131), managed to control infection more efficiently compared to the other groups, showing the potential effect of this peptidomimetic against the *P. berghei* strain. The *P. yoelii* strain had virulence features which were stronger that those displayed by *P. berghei*; animals challenged with the former strain thus handled higher parasitemia percentages. The effect of passively transferred antibodies in mice managed to control infection caused by *P. yoelii* in a similar way to that detected in the first case (Fig. 10a, b). As in the first case, animals treated with isotonic saline solution had lower parasitemia percentages, but died during a shorter period of time than the remaining study groups. All groups studied were challenged twice, the first time by intravenous administration and the second time intraperitoneally. Figure 11 displays the percentage of animal survival after being Ig passively administered.

Is should be stressed that all animals in the study groups had 90–100 % protection during the first 20 days of the in vivo assay during which the groups were experimentally challenged with *P. berghei,* and the first 12 days after experimental challenge of groups with *P. yoelii*. This is why it was decided to induce fresh infection (reinfection) intraperitoneally with twice the parasite load. As demonstrated, treated animals were able to efficiently control malaria infection until the passively administered antibody had decreased its concentration; thus, boosting with the same dose of immunoglobulin helped to maintain the protective effect. Parasitemia percentages measured after malaria in vivo infection of mice with *Plasmodium berghei* and *Plasmodium yoelii* passively administered with specific doses of monoclonal antibodies raised against site-directed designed pseudopeptides were as follows: mice groups administered mAbs against both ψ-128 and ψ-130 pseudopeptides and challenged with lethal doses of *Plasmodium berghei* showed parasitemia levels of about 0–0.10 % until 20 days after the first i.v challenge with the exception of groups 1 and 8. Group 1 was administered isotonic saline solution and group 8 was transferred with polyclonal antibodies against HPV virus-like particles, with both groups displaying parasitemia levels closer to 10 % at day 5 (Fig. 10a, b). After a second i.p more lethal challenging, parasitemia levels of all surviving animals were effectively controlled in those antibody-administered animals, while animals belonging to control groups either became rapidly infected or died after 4 days of the second challenge.

Mice groups administered with mAbs against ψ-128 pseudopeptides and challenged with lethal doses of *Plasmodium yoelii* showed parasitemia levels of about 0–0.15 % until 15 days after the first i.v challenge with the exception of group 1, which was administered isotonic saline solution, displaying parasitemia levels closer to 5 % at day 5 (Fig. 10a, b). As above, after a second i.p more lethal challenging, parasitemia levels of all surviving animals were effectively controlled in those antibody-administered animals, while animals belonging to the control group became rapidly infected or died after 3 days of the second challenge. As experimentally observed, the *Plasmodium yoelii* strain was more infective than the *Plasmodium berghei* strain.

On the other hand, isotype-defined immunoglobulins possess neutralizing activity of the in vitro *Plasmodium falciparum* infection of human RBCs, in a dose-dependent manner, since at a very low concentration these were efficient invasion inhibitors as can be seen in Table 3 and discussed below. This fact once more demonstrates the potential use of these antibodies for controlling human malaria.

**Table 3:** In vitro invasion inhibition of *Plasmodium falciparum* to human RBC by antibodies against the ψ-130 (^30^F–I^31^) pseudopeptide

	Sample	Dilution	Invasion %	Invasion inhibition % ± SD
Controls	RBCs		1	99 ± 0.012
	Parasitized RBCs		100	0 ± 0.036
	Chloroquine 1.85 mg/ml	01:27	18	82 ± 0.010
	Chloroquine 0.93 mg/ml	01:54	14	80 ± 0.015
	EGTA 1.9 mg/mL		31	69 ± 0.010

Ig pool (ionic strength) (mil)	μg/ml	Dilution	Invasion %	Invasion inhibition % ± SD
50	19.05	1/2	16	84 ± 0.046
		1/4	82	18 ± 0.056
		1/8	97	3 ± 0.012
100	56.92	1/2	81	19 ± 0.026
		1/4	101	−1 ± 0.059
		1/8	101	−1 ± 0.059
500	36.13	1/2	9	91 ± 0.021
		1/4	18	82 ± 0.056
		1/8	64	36 ± 0.047

## Discussion

### Why a peptide bond should be modified in target antigens

Nature-made amide bonds have a specific purpose, which is to join two amino acids in a very simple but smart way. Polypeptides are thus composed of amino acids covalently bonded to each other like wagons on a train and so the peptide bond is the “hook” that connects both building blocks called amino acids. Such a molecular hook becomes established once the carbonyl group belonging to the carboxylic primary function of a given amino acid is attacked by an available electron pair belonging to the primary amino function of another neighbor amino acid. Inside the cells, peptide bonds are formed within ribosomes in a controlled process called translation. Either, a natural ribosomal or a solid-phase process that origins a newborn peptide or protein is completed once a polypeptide chain is assembled by bonding the genome coded twenty *N*-α-amino-acids through peptide bonds.

This precise and well-designed molecular architecture allows establishing many proteins as required by living systems, always under a systematic genetical control.

However when the battle for survival between human pathogens and their target cells are directed to given molecules such as cell receptors, pathogens can use potent and unique molecular mechanisms able to hydrolyzing peptide bonds. Such molecular weapons are known as peptidases, a kind of proteases having specific substrates and thermodynamics for optimal activity. Thus, most pathogens have adopted these molecular mechanisms for either attacking human cells or destroying specific proteins and antibiotics and so ensuring their survival. These are only a couple of reasons why a peptide bond should be substituted. On the other hand, our laboratory is working in the hypothesis that parasitic human pathogens such as *Plasmodium falciparum* employ molecular evasion mechanisms of the host immune system, based on modulating a specific 3D structure on its own proteins and activating novel molecular pathways for invasion of red blood cells (RBCs). This later possible evasion mechanism could be blocked by mimicking those specific modulated 3D structures of parasite proteins by employing novel compounds so-named peptide mimetics and pseudopeptides able to induce site-directed antibodies having neutralizing properties of a malaria infection.

### Two different specific peptide bond substitutions in the same antigen lead to two well-defined peptide mimetics

The site-directed design of peptide mimetics from amide-reduced pseudopeptides using native sequence encoded 4044 (shown to be poorly immunogenic and non-protection inducing against malaria in previous studies and the present one) led to obtaining two powerful pseudopeptides which were able to induce a humoral immune response mediated by antibodies specifically recognizing two groups of MSA-2 antigen-derived proteins. The versatility of modifying peptide bonds between specific adjacent residues (involving two different residues, such as ^30^Phe-Ile^31^ and ^31^Ile-Asn^32^) when used as immunogens have induced two groups of antibodies exhibiting strong and specific reactivity; the first group induced by ψ-130 (^30^Phe-Ile^31^) recognized a 37.90 kDa band having relative mobility and the second group of antibodies induced by ψ-128 (^31^Ile-Asn^32^) specifically recognized two bands having 34.21 kDa and 30.54 kDa relative molecular weights. These isotype-defined monoclonal antibodies, derived from a hybridoma producing IgM isotype immunoglobulins through in vitro immunization, had neutralizing in vivo infection properties when passively transferred to BALB/c mice experimentally challenged with lethal doses of *Plasmodium berghei* and *Plasmodium yoelii,* in a double-challenge administered by two different routes. For the first time, we present some evidence about the existence of MSA-2 protein precursors or orthologous proteins present in the cytosol of two rodent *Plasmodium* species, *P. berghei* and *P. yoelii*. These antibodies allowed us to redefine the N-terminal MSA-2 epitope as being the eight-residue sequence ^25^KYSNTFIN^32^.

The well-defined molecular origin and background of the obtained isotype-defined immunoglobulins’ in vitro functional activity, demonstrated through infection assays in controlled cultures as well as the evidence reported in this study, led to proposing these reduced amide peptide mimetics as potential components for inclusion in the formulation of a sub-unit-based antimalarial vaccine and their induced antibodies as potential immunotherapeutic agents which could help to manage this disease.

### A pathway toward immunotherapy in infectious diseases

The site-directed design of peptide mimetics based on this and previously reported *Plasmodium* surface antigens will facilitate assessing the data reported in this study and constitute new molecular tools to be applied in the clinical management of malaria, as well as other transmissible diseases. Fab/F(ab)_2_′ immunoglobulin fragments or the entire highly reactive antibody could be further employed as a shock therapy in malaria endemic areas where infections could be controlled by intravenously administering antibodies. As demonstrated in this work, mice have controlled and resolved an experimental malaria infection after being passively transferred with specific peptide-mimetic-induced antibodies. On the other hand, in vitro antibody isotype switching strategies can be successfully used to ensure obtaining blocking neutralizing antibody in cell culturing, then desirable isotype antibodies can be purified, detoxified and properly formulated. In the present work two important clue issues have to be taken into account: first as proposed herein, a strategy for in vitro immunoglobulin switching was successfully allowed by hybridoma stimulation with a controlled dose of the antibody inducer pseudopeptide; and secondly bearing in mind that such isotype-defined antibodies were induced against specific peptide-bond isostere surrogates designed in a site-directed manner, this rationale could explain these antibody inhibitory effect on experimental malaria infection. As observed in Table 3, isotype-defined immunoglobulins induced against the ψ-130 pseudopeptide eluted at both 50 mM and 500 mM ionic strengths were able to inhibit *Plasmodium falciparum* to human RBCs in a dose-dependent manner in an average concentration of 40 μg/mL from 84 % ± 0.046 to 3 % ± 0.012 in the first case and from 91 % ± 0.021 to 36 % ± 0.047 in the second, evidencing a stronger inhibitory activity in the latter. Remarkably, immunoglobulins eluted at a 100 mM ionic strength did not have an inhibitory effect on the human in vitro malaria infection.

The analysis of the newly modified molecular structure, in which an isostere peptide bond was introduced for substituting a nature made one, secondary structure elements present in the native molecule are switched with other elements able the molecule to reach energy states that facilitate its interaction with antigen presenting molecules of a HLA and TCR provoking a particular immune response based on antibody production, these having specific neutralizing properties by using non-conventional mechanisms for blocking parasites. Mimicking molecules containing high energy with peptide mimetics such as reduced amide pseudopeptides which possess four sp3 tetrahedral hybridized bonds replacing the three sp2 planar of a normal peptide bond introduces new freedom degrees into the molecule and produces dramatic changes in the entire molecular structure. Bearing in mind that structurally modulated immunogens induces specific and site-directed antibodies able to neutralize pathogens in a specific manner, such compounds could be postulated to be new and useful for novel malarial vaccine formulations.
